# Genetic basis and adaptation trajectory of soybean from its temperate origin to tropics

**DOI:** 10.1038/s41467-021-25800-3

**Published:** 2021-09-14

**Authors:** Lidong Dong, Chao Fang, Qun Cheng, Tong Su, Kun Kou, Lingping Kong, Chunbao Zhang, Haiyang Li, Zhihong Hou, Yuhang Zhang, Liyu Chen, Lin Yue, Lingshuang Wang, Kai Wang, Yongli Li, Zhuoran Gan, Xiaohui Yuan, James L. Weller, Sijia Lu, Fanjiang Kong, Baohui Liu

**Affiliations:** 1 Innovative Center of Molecular Genetics and Evolution, School of Life Sciences, Guangzhou University, Guangzhou, China; 2 The Innovative Academy of Seed Design, Key Laboratory of Soybean Molecular Design Breeding, Northeast Institute of Geography and Agroecology, Chinese Academy of Sciences, Harbin, China; 3 Soybean Research Institute, National Engineering Research Center for Soybean, Jilin Academy of Agricultural Sciences, Changchun, China; 4 State Key Laboratory of Crop Stress Adaptation and Improvement, School of Life Sciences, Henan University, Kaifeng, China; 5 School of Computer Science and Technology, Wuhan University of Technology, Wuhan, China; 6 School of Natural Sciences, University of Tasmania, Hobart, Tasmania Australia

**Keywords:** Evolutionary genetics, Agricultural genetics, Genetic variation, Plant domestication

## Abstract

Soybean (*Glycine max*) serves as a major source of protein and edible oils worldwide. The genetic and genomic bases of the adaptation of soybean to tropical regions remain largely unclear. Here, we identify the novel locus *Time of Flowering 16* (*Tof16*), which confers delay flowering and improve yield at low latitudes and determines that it harbors the soybean homolog of *LATE ELONGATED HYPOCOTYL* (*LHY*). *Tof16* and the previously identified *J* locus genetically additively but independently control yield under short-day conditions. More than 80% accessions in low latitude harbor the mutations of *tof16* and *j*, which suggests that loss of functions of *Tof16* and *J* are the major genetic basis of soybean adaptation into tropics. We suggest that maturity and yield traits can be quantitatively improved by modulating the genetic complexity of various alleles of the *LHY* homologs, *J* and *E1*. Our findings uncover the adaptation trajectory of soybean from its temperate origin to the tropics.

## Abstract (web summary)

How soybean, a temperate origin crop, adapted to a tropical environment remains unclear. Here, the authors report *Tof16*, an ortholog of *LHY*, and the previously identified *J* locus, control soybean yield under short-day condition and loss of function of these two genes contributes to the adaptation to tropics.

## Introduction

Soybean (*Glycine max* [L.] Merr.) is one of the most economically important leguminous crops, as it provides more than one-quarter of the world’s protein for human and animal consumption^1^. In 2020/2021, global soybean production was 362 million tons; soybean production in low latitude regions (Brazil, Argentina, and India) comprised 53.45 percent of the total worldwide production (United States Department of Agriculture, 2020). Therefore, improving soybean productivity in the tropics will have a great effect on fulfilling the demands of the growing worldwide population.

Cultivated soybean was domesticated from its wild progenitor (*Glycine soja* Sieb. & Zucc.) in temperate regions of China ~5000 years ago^2–4^. This facultative short-day (SD) plant is extremely sensitive to photoperiod^5^. This high sensitivity to photoperiod dramatically impedes the improvement of soybean productivity in the tropics. For instance, cultivars that are adapted to temperate regions flower early and produce extremely low grain yields when grown in low-latitude regions^6–8^. The long-juvenile (LJ) trait, which delays flowering and enhances grain yields under SD conditions, was first introduced into soybean cultivars in Brazil in the 1970s, which expanded soybean cultivation into the tropics^7–10^. Only two loci, *J* and *E6*, have been reported to control the LJ trait; both contain the same ortholog of *Arabidopsis thaliana EARLY FLOWERING 3*^10–13^. However, the genetic basis and trajectory of adaptation of soybean to low latitudes remain largely unknown.

In the current study, we identify a novel locus that controls flowering time and yield in soybean under SD conditions. This locus, *Tof16*, harbors a *LATE ELONGATED HYPOCOTYL* (*LHY*) gene, as reveals by whole-genome resequencing and genome-wide association studies (GWAS) and by positional cloning of quantitative trait loci (QTL). We confirm that four *LHY* homologs redundantly control flowering time and yield. We also demonstrate that *Tof16* and *J* additively but independently control soybean flowering. Both Tof16 and J directly bind to the promoter of *E1*, which encodes a core flowering suppressor, to repress its transcription. Interestingly, weak mutant alleles for both *tof16* and *j* are initially selected, and additional null alleles are subsequently acquired in addition to these weak alleles and further selected, suggesting that stepwise selection of natural mutations of both genes occur during the adaptation of soybean from temperate regions to the tropics. By modulating the combinations of mutations of *LHY* homologs or combinations of different alleles of *Tof16, J*, and *E1*, maturity and yield are altered in a quantitative manner. Our findings uncover the genetic and genomic basis for the adaptation of soybean to low-latitude regions and provide a new approach for precise breeding for improve soybean productivity in the tropics.

## Results

### Resequencing of soybean accessions from low latitudes

To investigate the genomic basis for the natural variation in soybean adaptation to low latitudes, we conducted whole-genome resequencing of a panel of 329 soybean accessions collected from 15 countries and covering all soybean subgroups in which 165 accessions are from in low-latitude regions (Supplementary Fig. 1a and Supplementary Data 1). Using the whole-genome single-nucleotide polymorphism (SNP) marker set, we performed phylogenetic analysis and principal component analysis (PCA) to quantify the population structure of these 329 soybean accessions. These analyses clearly classified the accessions into three groups: wild soybeans, landraces, and improved cultivars, and four main regions: China, Southeast Asia, South Asia, and South America (Supplementary Fig. 1b, c, Supplementary Fig. 2 and Supplementary Data 1). Consistent with previous findings^14,15^, the decay of linkage disequilibrium (*r*^2^) with the physical distance between SNPs occurred in all three groups (Supplementary Fig. 1d).

### Identification of the *Tof16* locus

**Fig. 1:**
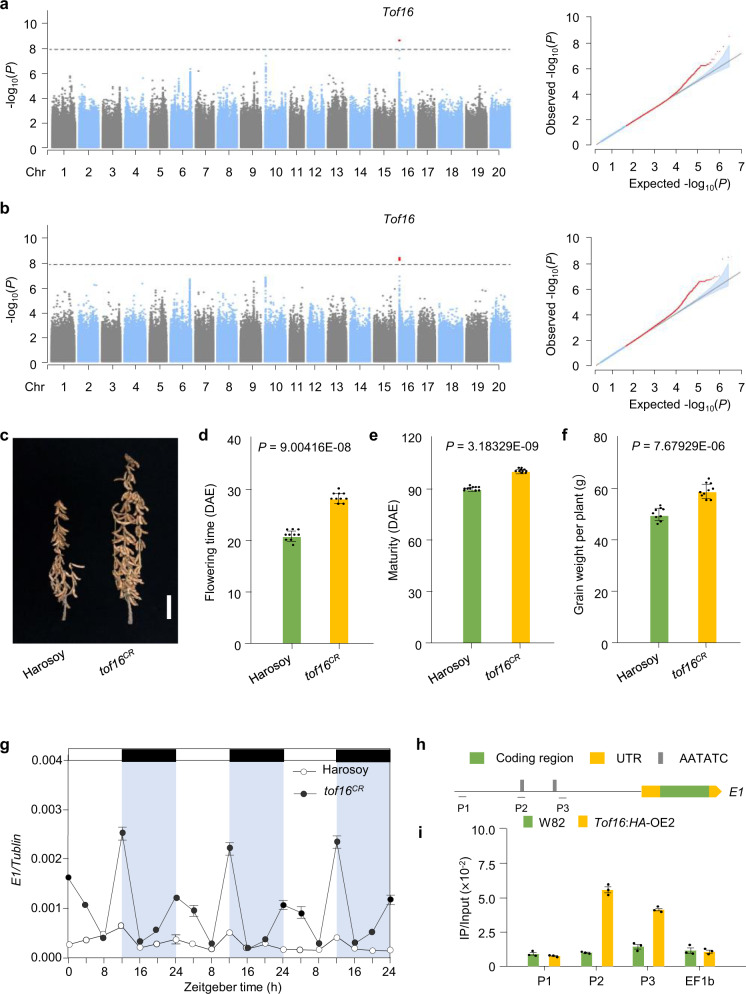
Identification of *Tof16*. **a**–**b** GWAS scan for flowering time (R1 stage) using data from the 329-accession panel grown over the 2018 (**a**) and 2019 (**b**) field seasons in Guangzhou, China. **c** Phenotypes of *tof16* mutation and wild-type Harosoy under SD (12 h light/12 h dark) conditions. Scale bar, 10 cm. **d** Flowering time. **e** Time to maturity. **f** Grain yield per plant of *tof16*^*CR*^ mutation and Harosoy. All data were given as mean ± s.e.m. (*n* = 10 plants), the value of each plant was represented by a dot. One-tailed Student’s *t*-test was used to generate the *P* values. **g** Diurnal variation in transcript levels of in wild-type Harosoy and *tof6*^*CR*^ mutant under SD. All data were given as mean ± s.e.m. (*n* = 5 plants). **h** Location of the AATATC-motif in *E1* gene promoter. **i** ChIP-qPCR results demonstrated that Tof16 directly bound to the promoter of *E1* gene. Values are mean ± s.e.m. (*n* = 3 biologically independent samples), the value of each replication was represented by a dot. Source data underlying Fig. 1d–g and  i are provided as a Source Data file.

Using a linear mixed model for GWAS of the panel of 329 accessions, we identified one consistent significantly associated locus on chromosome 16 (*P* < 1.13 × 10^−8^) controlling flowering time under natural SD conditions in both 2018 and 2019 in Guangzhou. (Fig. 1a, b). This GWAS peak is consistent with the SD flowering QTL on chromosome 16 that we previously identified using two F_2_ segregation populations, PI591429 × PI628930 and PI240664 × BR121^16^. These analyses across multiple biparental and natural populations indicate that variation at this locus (hereafter referred to as *Time of Flowering 16*, *Tof16*) is widespread and substantially contributes to the control of flowering time under SD conditions.

### *Tof16* encodes LHY1a

We generated a large (*n* = 2418) inbred F_6_ population from the PI591429 × PI628930 cross for fine-mapping of the *Tof16* locus by recurrent selection for heterozygosity at *Tof16* from the F_2_ to F_5_ generations. Analysis of this population located *Tof16* within a 120-kilobase (kb) region harboring 12 genes based on the Williams 82 reference genome^17^ (Supplementary Fig. 3a, b and Supplementary Table 1). We cloned and sequenced all 12 of the predicted genes in the two parents; of these, the sequence of the circadian clock gene *LHY1a* (*Glyma.16G017400*) differed between the two parents (PI591429 and PI628930) (Supplementary Fig. 3b). The late flowering parent PI628930 harbored two SNPs predicted to cause a gain of a stop codon, resulting in premature termination of translation after 159 amino acids in the 750-amino-acid LHY1a protein (Supplementary Figs. 3b and 4). We also sequenced the coding region of *LHY1a* in PI240664 and BR121, finding that in PI240664, this gene harbored one SNP predicted to convert a serine into a cysteine, which is a conserved site in leguminous plants (Supplementary Figs. 4 and 5). The presence of different mutations in these two lines suggested that the *LHY1a* gene was a strong candidate for the *Tof16* locus.

To validate whether *LHY1a* is the causative gene of the *Tof16* locus, we generated loss-of-function mutations of *LHY1a* (named *tof16*^*CR*^) in the Harosoy background using CRISPR/Cas9-mediated gene editing and evaluated the phenotypes of the mutants vs. wild-type Harosoy (Supplementary Fig. 6). The *tof16*^*CR*^ plants showed significantly delayed flowering time and maturity, altered yield-related traits, and improved overall grain yield relative to Harosoy (Fig. 1c–f, Supplementary Fig. 7a–d). These results confirm the notion that *LHY1a* is the causative gene of the *Tof16* locus and that two natural mutations arose in PI628930 and PI240664 (hereafter referred to as *tof16*-*1* and *tof16*-*2*, respectively).

To examine the specific effects of the *Tof16* locus, we also compared the phenotypes of two F_7_ near-isogenic lines (NILs) carrying either the functional *Tof16* allele (NIL-*Tof16*) or the non-functional *tof16*-*1* alleles (NIL-*tof16-1* or NIL-*tof16-2*) in different genetic backgrounds. NIL-*tof16*-*1* and NIL-*tof16*-*2* showed significantly delayed flowering time and maturity (Supplementary Fig. 8a–c, e–g), along with increased plant height, node number, pod number, branch number (Supplementary Fig. 7e–l), and grain yield compared to the functional NILs (Supplementary Fig. 8d, h), confirming the notion that the *tof16*-*1* and *tof16*-*2* alleles delay flowering and greatly enhance grain yields in soybean under SD conditions. Therefore, like *J*, *Tof16* also functions as a flowering enhancer; the loss of function of both genes might have contributed to the adaptation of soybean to the tropics.

### *Tof16* is genetically dependent on the legume-specific flowering repressor *E1*

E1 plays a central role in photoperiodic flowering by repressing the expression of two key *FT* homologs, *FT2a* and *FT5a*^14,18^. There are three major natural alleles of *E1* in soybean: *E1*, *e1*^*as*^, and *e1*^*fs*^; *e1*^*as*^ is a weak mutant allele and *e1*^*fs*^ is a null functional allele^18^. To examine the genetic interaction of *Tof16* and *E1*, we developed a NIL set for *E1*/*Tof16*, *E1*/*tof16*^*CR*^, *e1*^*as*^/*Tof16, e1*^*as*^*/tof16*^*CR*^, *e1*^*fs*^/*Tof16*, and *e1*^*fs*^/*tof16*^*CR*^ in the Harosoy background and performed phenotypic analysis. The *tof16*^*CR*^ allele delayed flowering and maturity in both the *E1* and *e1*^*as*^ genetic backgrounds, but the effect was weaker in the *e1*^*as*^ background. By contrast, the effect of *Tof16* on flowering was completely eliminated in the *e1*^*fs*^ null functional background (Supplementary Fig. 9a–c), implying that the full effect of *Tof16* on flowering depends on *E1*.

We evaluated the effect of *Tof16* on the transcriptional regulation of *E1*, *FT2a*, and *FT5a* under SD (12 h light/12 h dark) conditions using *tof16*^*CR*^ and Harosoy or independent NIL pairs for each locus. As expected, *E1* was expressed at higher levels in *tof16*^*CR*^ (Fig. 1g), and *FT2a* and *FT5a* were expressed at lower levels in this line compared to Harosoy (Supplementary Fig. 10a, b). A similar result was obtained for the NILs. These results indicate that functional alleles of *Tof16* repress *E1* expression and increase *FT2a* and *FT5a* expression relative to the mutant alleles (Supplementary Figs. 10c, d and 11).

To further explore the molecular nature of the relationship between *Tof16* and *E1*, we determined whether Tof16 directly binds to the promoter of *E1* in vivo. We generated transformants overexpressing *Tof16-6HA* in the Williams 82 background and subjected them to chromatin immunoprecipitation (ChIP)-qPCR assays (Supplementary Fig. 12). Tof16 directly associated with the *E1* promoter regions that contained AATATC motif (a part of the EE motif, Fig. 1h, i). These results consist with our previous finding that Tof16 protein could bind to AATATC motif in the *E1* promoter in vitro^14^. Taken together, these results indicate that Tof16 enhances early flowering and maturity by direct associating with the *E1* promoter to suppress *E1* expression, thus releasing *FT2a* and *FT5a* transcription.

### Four *LHY* homologs redundantly control soybean flowering and grain yield

**Fig. 2:**
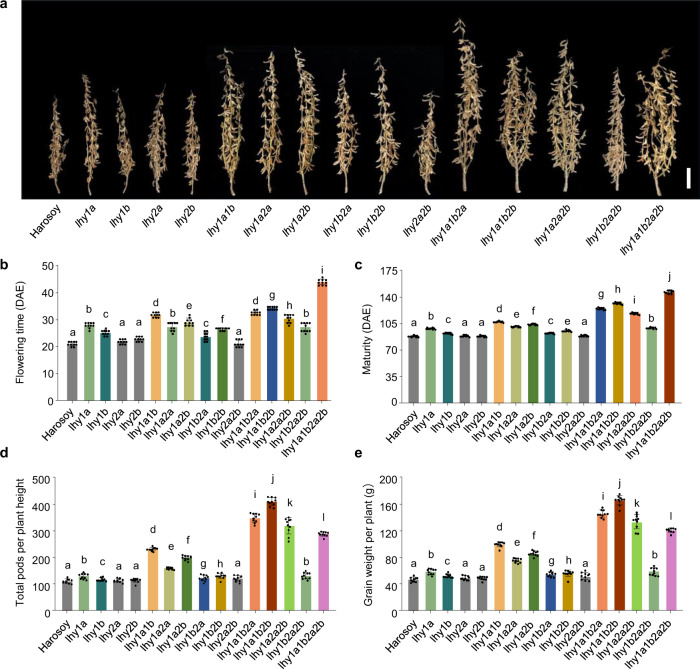
Redundancy among four *LHY* genes regulates soybean flowering time and yield under SD (12 h light/12 h dark) conditions. **a** Phenotypes of *lhy* mutants. Scale bar, 10 cm. **b** Flowering time. **c** Time to maturity. **d** Total pods per plant height. **e** Grain yield per plant. All data were given as mean ± s.e.m. (*n* = 10 plants), the value of each plant was represented by a dot. The presence of the same lowercase letter above the histogram bars in (**b**–**e**), denotes nonsignificant differences across the two panels (*P* > 0.05). One-way ANOVA was used to generate the *P* values. Source data underlying Fig. 2b–e are provided as a Source Data file.

The soybean genome contains four *LHY*/*CCA1* homologs (*LHY1a*, *LHY1b*, *LHY2a*, and *LHY2b*)^14,19^ and its amino acid sequences were high homology (Supplementary Fig. 5). Since we demonstrated that *Tof16/LHY1a* controls flowering time, maturity, and grain yield in soybean, we asked whether the other *LHY* family members also control these soybean traits and whether these homologs could potentially be used for agricultural applications. We crossed the *lhy1a/1b/2a/2b* quadruple mutants with wild-type Harosoy and obtained all 15 homozygous mutational combinations of *LHY* (Fig. 2a). We examined the phenotypic differences of the mutants grown in fields in Guangzhou under natural SD conditions. Among the single mutants, *lhy1a* and *lhy1b*, but not *lhy2a* or *lhy2b*, showed significantly delayed flowering time and maturity and improved overall grain yield relative to Harosoy (Fig. 2a–e, Supplementary Fig. 13a–d). All of the multiple mutants except the *lhy2c/2d* double mutants showed significantly delayed flowering time and increased grain yield in a quantitative manner compared to wild-type Harosoy (Fig. 2a–e, Supplementary Fig. 13a–d). Strikingly, the *lhy1a/1b/2b* triple mutants exhibited the best architecture and highest grain yield but shorter flowering time and earlier maturity compared to the *lhy1a/1b/2a/2b* quadruple mutants under natural SD conditions (Fig. 2a–e, Supplementary Fig. 13a–d). Furthermore, all mutants possessing the *lhy1a* (*tof16*) mutation had higher grain yields than those without this mutation, indicating that *lhy1a* plays a crucial role in controlling flowering time, maturity, and grain yield under SD conditions (Fig. 2a–e, Supplementary Fig. 13a–d).

To gain further insight into how *LHY* homologs control flowering time and grain yields under long-day (LD) conditions, we also evaluated the mutants in the field in Changchun under natural LD conditions. All mutants except *lhy2a*, *lhy2b*, and *lhy2a/2b* showed delayed flowering time and maturity compared to the wild type (Supplementary Fig. 14a–i). Due to the larger effect of *LHY1a* on flowering time, multiple mutants carrying the *lhy1a* mutation failed to mature, and it was difficult to harvest the seeds naturally until the end of the growing season (Supplementary Fig. 14a–i). However, unlike their performance under SD conditions, the *lhy1a* and *lhy1b2a2b* mutants exhibited the best architecture and improved grain productivity under natural LD conditions. These results suggest that *LHY* homologs have redundant but divergent functions in controlling flowering time, maturity, and grain yield in soybean under both SD and LD conditions. We further examined the expression of four *LHY* genes under LD and SD conditions, and found that the expression of four *LHY* genes were no significant difference under LD and SD conditions (Supplementary Fig. 15). We also tested the expression levels of *E1* in *lhy* multiple mutants, the result showed that the amount of *E1* correlated with flowering and maturity under LD and SD conditions, but not yield (Supplementary Figs. 16–19). Therefore, manipulating the combinations of these alleles and modulating the genetic complexity of the *LHY* homologs could help create the appropriate genotypes to maximize the adaptation and yield potential of soybean at different latitudes.

### *Tof16* and *J* additively control flowering time and grain yield

**Fig. 3:**
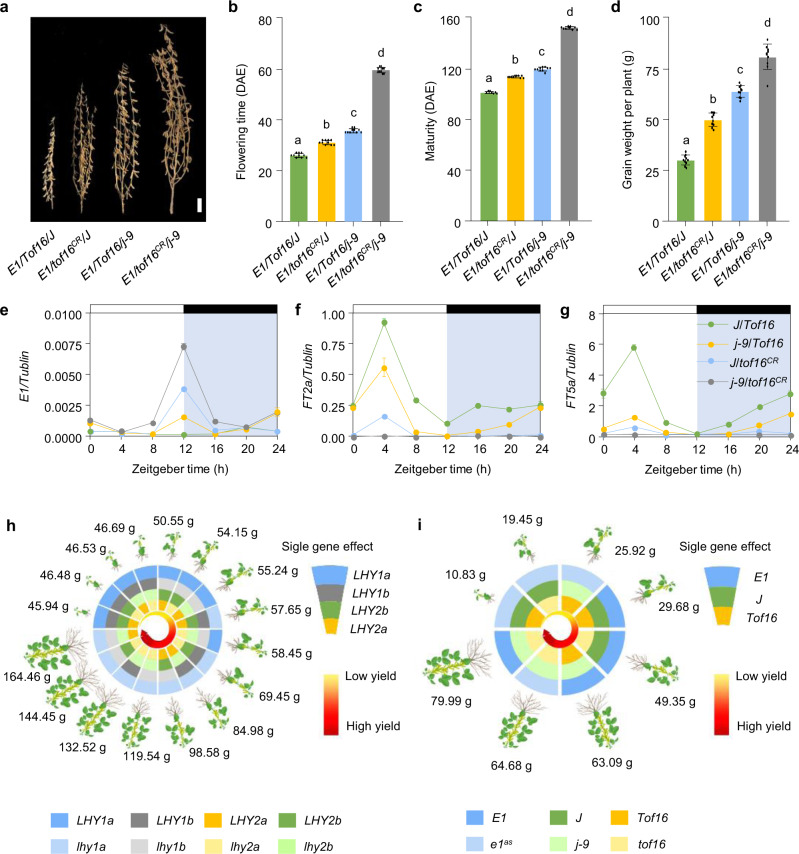
Genetic and regulatory interactions of *Tof16* and *J*, and model summarizing of combining natural or engineered alleles of *LHY* family and *J* improve soybean yield. **a** Phenotypes of NILs possessing different allelic combinations at *Tof16* and *J* in *E1* background under SD (12 h light/12 h dark) conditions. Scale bar, 10 cm. **b** Flowering time. **c** Time to maturity. **d** Grain weight per plant. All data were given as mean ± s.e.m. (n = 10 plants), the value of each plant was represented by a dot. The presence of the same lowercase letter above the histogram bars in (**b**–**d**) denoted nonsignificant differences across the two panels (*P* > 0.05). One-way ANOVA was used to generate the *P* values. **e**–**g** Diurnal variation in transcript levels of *E1* (**e**)*, FT2a* (**f**)*, FT5a* (**g**) in possessing different allelic combinations at *Tof16* and *J* in *E1* background under SD conditions. All data were given as mean ± s.e.m. (*n* = 5 plants). **h** Combining of various CRISPR/Cas9 generated mutants of *LHY* allows improve soybean adaption to tropic regions and yield. The value represents the average grain weight per plant in fields of Guangzhou under natural SD conditions. **i** Combining of natural or gene-edited of *Tof16* and *J* alleles in back ground *E1* or *e1*^*as*^ enhance soybean yield in tropic regions. The value represents the average grain weight per plant in fields of Guangzhou under natural SD conditions. Source data underlying Fig. 3b–g are provided as a Source Data file.

The adaptation of soybean to low latitude or tropical regions largely depends on the natural loss of function of the flowering enhancer *J*^12^. Therefore, it is critical to explore the genetic relationship between *Tof16* and *J*. We tested the reciprocal transcriptional regulation between *Tof16* and *J* using mutants or NIL sets of *tof16* or *j*. No mutual transcriptional regulation was observed between *Tof16* and *J* (Supplementary Fig. 20). To further explore the genetic interaction of *Tof16* and *J*, we developed two NIL sets for the four different homozygous allelic combinations at two loci from a cross between *tof16*^*CR*^*/E1* and NIL-*j*/*E1* or a cross between *tof16*^*CR*^*/e1*^*as*^ and NIL-*j/e1*^*as*^ in the Harosoy background and subjected them to phenotypic evaluation. The presence of a recessive allele of either *Tof16* or *J* delayed flowering and maturity and enhanced grain yield in both the *E1* and *e1*^*as*^ genetic backgrounds. However, the double recessive mutant *tof16 j* showed significantly later flowering, maturity, and higher grain yield than either the *j* or *tof16* single mutant in both the *E1* and *e1*^*as*^ genetic backgrounds (Fig. 3a–d, Supplementary Fig. 21a–l), suggesting that *Tof16* and *J* additively control flowering and grain yield in a genetically independent manner.

Consistent with this genetic effect, *E1* transcript levels were highest in NIL-*tof16*^*CR*^*/j*, followed by NIL-*Tof16/j* or NIL-*tof16*^*CR*^*/J*, and were lowest in NIL-*Tof16/J*. As expected, *FT2a* and *FT5a* showed the opposite expression pattern (Fig. 3e–g). These results, together with our previous findings^12^, suggest that the positive regulators of flowering *Tof16* and *J* both promote flowering, which depends on the function of *E1*. However, *Tof16* and *J* independently but additively regulate flowering time, maturity, and grain yield in soybean.

Based on these findings, we propose two possible methods for the quantitative improvement of flowering time and grain yield in soybean in tropical regions (Fig. 3h, i): (1) Due to the genetically redundant and divergent effects of *LHY* homologs, genotypes with a quantitative series of flowering time, maturity, and yield traits could be created by combining various CRISPR/Cas9-generated mutants (Fig. 3h). (2) Due to the genetic effects of *Tof16*, *J*, and *E1*, their various natural or artificial alleles could be combined, allowing another set of genotypes conditioning quantitative traits to be produced (Fig. 3i). How these genotypes could be selected or utilized remains to be explored and depends on the photoperiod or latitudinal environment.

### Stepwise selection of *Tof16* and *J* during soybean adaptation

**Fig. 4:**
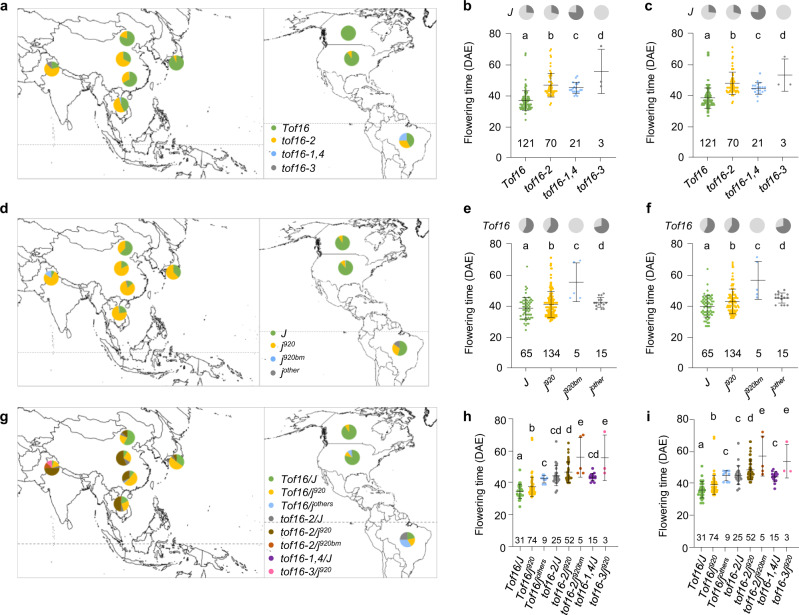
Geographical distribution of genetic diversity of *Tof16* and *J*. **a** Loss-of-function alleles of *Tof16* frequency is highly correlated with low latitude regions. Data from 1624 diversity panels. **b**–**c** Flowering time(R1) variations in 329 accessions possess *Tof16* and *tof16* alleles in Guangzhou 2018 (**b**), and Guangzhou 2019 (**c**). Proportions of *j* alleles in upper pie chart. Dark gray represents *J* alleles and light gray represents *j* alleles. **d** Loss-of-function alleles of *J* frequency is highly correlated with low latitude regions. Data from 1624 diversity panels. *j*^*920*^ represents *j-11* allele including *J-SNP*^*G920T*^*. j*^*920bm*^ represents SNP920-based mutations including *j-1, j-3*, *j-6* and *j-10* mutational alleles. *j-others* represents *j* mutations other than *j*^*920bm*^. **e**–**f** Flowering time(R1) variations in 329 accessions possess *J* and *j* alleles in Guangzhou 2018 (**e**), and Guangzhou 2019 (**f**). Proportions of *tof16* alleles in upper pie chart. Dark gray represents *Tof16* alleles and light gray represents *tof16* alleles. **g** Loss-of-function *Tof16* and *J* improve soybean adaptation to low latitudes. Data from 1624 diversity panels. **h**–**i** Flowering time variations of eight allelic combinations of *Tof16* and *J* in Guangzhou 2018 (**h**), and Guangzhou 2019 (**i**). The presence of the same lowercase letter above the histogram bars in (**b**, **c**, **e**, **f**, **h**, **i**) denotes nonsignificant differences across the two panels (*P* > 0.05). One-way ANOVA was used to generate the *P* values. All data were given as mean ± s.e.m. The value of each plant was represented by a dot. Source data underlying Fig. 4b, c, e, f, h, and i are provided as a Source Data file.

Our findings indicate that the positive regulators of flowering *Tof16* and *J* play essential roles in the adaptation of soybean to SDs and yield development. To gain insight into the evolutionary trajectory of soybean adaptation from its temperate origin to the tropics, we examined the genomic variations in the *Tof16* and *J* coding sequences in 1624 resequenced soybean accessions, including 1295 previously described accessions^14,20^ and the 329-accession panel used in the current study. We identified 15 unique high-confidence haplotypes in *Tof16*. In addition to haplotypes H10 (*tof16-1*) and H11 (*tof16-2*), we identified two novel loss-of-function haplotypes: H1 (named *tof16-3*) and H8 (named *tof16-4*) (Supplementary Fig. 22a, Supplemental Data 2). Analysis of haplotype origin indicated that the *tof16-2* allele (H11, *Tof16-SNP*^*A1276T*^) first occurred in wild soybeans originating in the Huanghui region and were subsequently domesticated into landraces in areas where soybean domestication occurred (Fig. 4a, Supplementary Fig. 22b). Following domestication and during the adaptation of soybean to low latitudes, H11 (*tof16-2*) was intensively selected in the accessions that adapted to low-latitude regions, suggesting that this haplotype plays critical roles in soybean adaptation to the tropics (Fig. 4a). Interestingly, H10 (*tof16-1*) and H8 (*tof16-4*) originated from H11 (*tof16-2*), suggesting that loss-of-function alleles of *tof16-2, tof16-1*, and *tof16-4* were under stepwise selection during adaptation to low latitudes. However, H8 (*tof16-3*) originated from H1 and only occurred in India and Nepal, while *tof16-1* and *tof16-4* mainly occurred in Brazil, indicating that unique loss-of-function alleles of *tof16* were selected in different regions to enhance the adaptation of soybean (Fig. 4a, Supplementary Fig. 22b).

To further validate the functional significance of *tof16-1*, *tof16-2, tof16-3*, and *tof16-4*, we performed a transient transfection assay in *Arabidopsis thaliana* protoplasts. *tof16-2* partial impaired the ability of Tof16 to repress the expression of *E1*, but *tof16-1*, *tof16-4*, and *tof16-3* completely impaired this ability (Supplementary Fig. 23). These results imply that partial loss of function of *tof16-2* (standing variations from soybean in the central area of origin) was first selected during soybean adaptation to the tropics but was not sufficient for full adaptation. In this genetic background, null alleles of *tof16-1* and *tof16-4* occurred and were further selected for better adaptation to the tropics. Population genetic association analysis of flowering time in the 329-accession panel showed that *tof16-2* flowered the latest, followed by *tof16*-*3*, *tof16-1,4*, and *Tof16* in both 2018 and 2019 (Fig. 4b, c). The unexpected finding that the weak functional allele *tof16-2* flowered later than the loss-of-function alleles *tof16-1*, *tof16-4*, and *tof16-3* could be explained by the interference of the loss of function of *j* (Fig. 4b, c). Taken together, these data suggest that selection at *tof16-1*or *tof16-4* and *tof16-2* arose in a stepwise manner. *Tof16* loss-of-function alleles independently originated and were selected in two important soybean planting areas (Brazil and India) in the tropics.

We screened for natural variation of the *J* coding sequence in the same 1624-accession panel. In total, 28 haplotypes were identified, including seven distinct loss-of-function alleles and two weak loss-of-function alleles. These haplotypes included the previously reported alleles *j-1*, *j-2*, *j-3*, *j-4*, *j-6*, *j-8* (including *j-8-1* and *j-8-2*), and *j-9* (*e6*)^12,13^ and the newly discovered alleles haplotype H18 (named *j-10*) and haplotype H21 (named *j-11*) (Supplementary Fig. 24a, Supplementary Data 2). Like *tof16-2*, haplotype origin network analysis indicated that haplotype H21 (*j-11*, a *SNP*^*G920T*^ resulting in an amino acid substitution) also first occurred in wild soybean originating from Huanghuai and was later was domesticated into landraces, but this allele was substantially selected in accessions grown in low-latitude regions (Fig. 4d, Supplementary Fig. 24b). Furthermore, loss-of function alleles *j-1*, *j-3*, *j-6*, and *j-10* all occurred in the tropics and originated from haplotype H21 (*j-11*), indicating that, similar to *tof16*, stepwise selection of the weak allele of *j-11* and other loss-of-function alleles of *j* occurred during the adaptation of soybean to the tropics (Fig. 4d, Supplementary Fig. 24b).

Transient transfection assays also demonstrated that *j-11* partially impaired the ability of J to repress the expression of *E1* (Supplementary Fig. 25), indicating that H21 (*j-11*, SNP *j*^*920*^) is a weak loss-of-function allele that might contribute to adaptation to low latitudes. We further classified all haplotypes of *J* into four groups, *J*, *j*^*920*^, *j*^*920bm*^ (SNP920-based mutations, including the *j-1*, *j-3*, *j-6*, and *j-10* mutant alleles), and *j*^*other*^ (*j* mutations other than *j*^*920bm*^) and evaluated their associations with flowering in the 329-accession panel. *j*^*920bm*^ flowered the latest, followed by *j*^*other*^, *j*^*920*^, and *J* in both 2018 and 2019 (Fig. 4e, f). Taken together, our data suggest that like the selection at *Tof16*, the selection of *j*^*920bm*^ and *j*^920^ also arose in a stepwise manner.

### Selection of natural mutations of *Tof16* and *J* allowed soybean to move into the tropics

We firstly investigated that the distribution of the various natural alleles of *E1*, *Tof16*, and *J* in tropical soybean accessions. We found that all of the tropical accessions harbor the dominant *E1* allele (Supplementary Data 3), and the frequency of the *tof16-2* allele (26.75%) and *j-11* allele (47.42%) were highly variable in tropical accessions (Supplementary Tables 2 and 3). To further explore the contributions of mutations of *Tof16* and *J* to the adaptation of soybean to low latitudes, we grouped eight allelic combinations (*Tof16/J*, *Tof16*/*j*^*920*^, *Tof16*/*j*^*others*^, *tof16-2*/*J*, *tof16-2*/*j*^*920*^, *tof16-2*/*j*^*920bm*^, *tof16-1,4*/*j*^*920*^, *tof16-3*/*j*^*920*^) and examined the geographic distributions of the eight *Tof16/J* allelic combinations in the 1624 accessions covering all latitudes. Accessions carrying loss-function-of *Tof16* alleles were enriched in Brazil, but accessions carrying loss-function-of *J* alleles were enriched in Southern China, Southeast Asia, and Brazil (Fig. 4g), suggesting that the selection of mutations of *tof16* and *j* might have occurred independently. Interestingly, by extracting the 165 accessions from low latitude, we found that more than 80% accessions harbor the loss of functions of *Tof16* and *J* suggest the two genes are the major genetic forces to drive soybean adaptation from temperate into tropics (Supplementary Data 4, Supplementary Table 4). By contrast, most accessions adapted to high-latitude regions such as Northern China, the United States, and Canada, where early flowering is required, carried the full functional alleles of *Tof16* and *J* (Fig. 4 a, d, g), suggesting that the mutations of these two genes helped soybean move from its temperate origins into the tropics.

Finally, to further explore the functional significance of *tof16* and *j*, we examined their association with flowering time in the 329-accession panel in two environments in Guangzhou (2018 and 2019). Accessions carrying two recessive alleles flowered significantly later than accessions carrying single recessive alleles *tof16* or *j*, and accessions carrying both functional alleles (*Tof16/J*) flowered earlier than the other three genotypic groups in all environments (Fig. 4h, i). These results further confirm the genetic additive effects of *Tof16* and *J* revealed from their interactions in NILs (Fig. 3b–d). Therefore, the selection of natural mutations of both genes has played substantial roles in expanding soybean cultivation from its temperate origins into the tropics and has facilitated the improvement of soybean adaptation and yield in the tropics.

## Discussion

Soybean production in low-latitude regions already accounts for approximately half of the world’s total production. Understanding the genetic and genomic basis of soybean adaptation to low latitudes will greatly facilitate the improvement of soybean productivity. In the current study, we identified the novel locus *Tof16*, which harbors the *LHY1a* gene, and determined that it contributes to geographical adaptation to low latitude regions and improves soybean yields. Consistent with our previous study^14^, we confirmed that *Tof16* depends genetically on the legume-specific flowering repressor *E1* and that Tof16 directly binds the promoter of *E1* to repress its transcription, which in turn releases its transcriptional suppression of two key soybean *FT* homologs (Fig. 1g, h and Supplementary Figs. 10 and 11).

The adaptation of soybean to the tropics also depends on selection for natural mutations of *J*, a homolog of the circadian clock gene *ELF3*. J also directly binds to the promoter of *E1* to repress its transcription^12^. A recent report indicates that J associates with two LUX homologs to form the evening complex, which binds to the *E1* promoter through the LUX MYB binding domain^21^. The two *LUX* homologs redundantly control soybean flowering, as the single mutants show no phenotypes but the *lux1 lux2* double mutant exhibits the same extremely late flowering under both SD and LD conditions, suggesting that the evening complex plays key roles in photoperiodic flowering and photoperiod sensitivity in soybean^21^.

Two other circadian clock genes, *Tof11* and *Tof12*, which are homologs of *PRR3*, play essential roles in soybean domestication and in cultivars adapted to high latitudes. Both Tof11 and Tof12 control flowering via *E1*, but they do this indirectly by inducing *E1* transcription by binding to the promoters of *LHY* homologs^14^. Collectively, these findings strongly suggest that the legume-specific transcription factor E1 plays critical roles in photoperiodic flowering, thus controlling adaptation and yield development in soybean. These findings also indicate that circadian clock genes act upstream of *E1* to regulate its transcription. Therefore, dissecting the functions and elucidating the genetic networks of circadian clock genes would increase our understanding of photoperiodic flowering and adaptation in soybean.

**Fig. 5:**
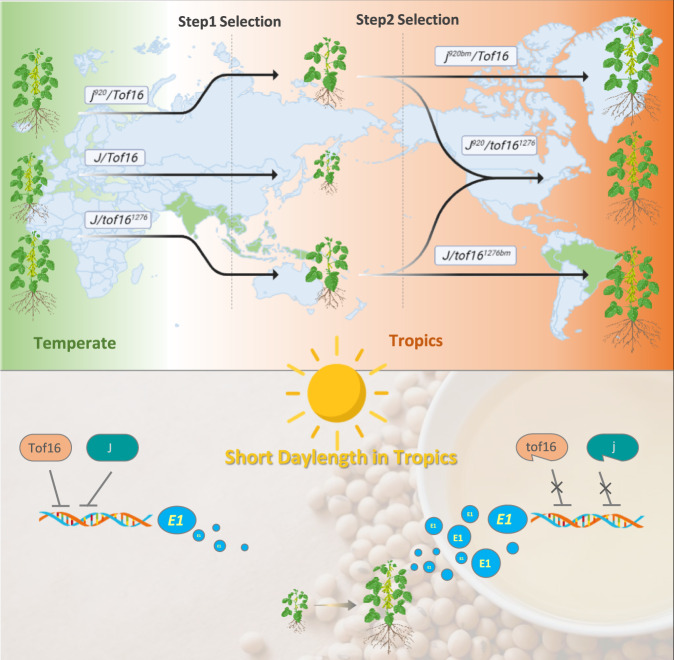
Evolutionary trajectory of *Tof16* and *J* during low latitude adaptation. Artificial stepwise selection on the various mutational alleles of *tof16* and *j* helps soybean gradually adapted into tropics from its temperate origins. Weak mutant alleles for both *tof16* and *j* were firstly selected, and additional null alleles were subsequently acquired in addition to these weak alleles and further selected. Tof16 and J proteins directly bind to the promoter of *E1* gene to suppress its transcription. When the protein function of Tof16 and J is damaged, the transcriptional suppression of *E1* is released, ultimately resulting in delay flowering and maturity and enhance grain yield.

Decoding the genomic basis of the natural variation of flowering genes is central to understanding crop adaptation and yield improvement. Loss-of-function alleles of *J* were successfully introduced into soybean cultivars in central-western Brazil, which subsequently enabled the expansion of soybean production to regions below 15° latitude and even as far as the equator^6,8,10^. Our analysis of the selection of natural variations of *Tof16* and *J* sheds light on how soybean adapted from its temperate mid-latitude origins to low latitudes. Both *tof16* and *j* (*tof16-2* and *j-11*) are relatively weak alleles encoding proteins with amino acid substitutions that occurred in wild soybean from the Huanghuai region in China, the center of origin of soybean (Fig. 4a, d, Supplementary Figs. 22–25). During domestication, these two alleles were retained and passed onto the landraces in this region (Fig. 5 and Supplementary Data 2). When the landraces disseminated into low-latitude regions, these two alleles were first selected due to their partial functional impairments, which delay flowering at low latitudes (Fig. 5). However, this delayed flowering was not sufficient to meet the requirements for complete adaptation and yield development. Consequently, more complete loss-of-function mutations occurred on top of the two earlier weak alleles and were intensively selected and incorporated into breeding programs to further improve plant adaptation and yield in the tropics (Fig. 4a–f).

The interesting stepwise selection process of soybean adaptation to the tropics was not identified previously due to the lack of available whole-genome sequences of large groups of accessions. Stepwise selection of flowering-time genes was also identified for *Tof12* and *Tof11* homologs during the adaptation of soybean to high latitudes. This led to the gradual loss of photoperiod sensitivity to facilitate growth during the short growing season and to synchronize the harvest^14^. Stepwise selection of different *cis*-regulatory variants in an *FT* homolog also occurred at different evolutionary times for local adaptation in maize^22^. A recent study also indicated that stepwise selection of a *CONSTANS LIKE* gene and a *Phytochrome A* gene facilitated the adaptation of common bean to high latitudes^23^. These findings suggest that the gradual selection of different genes or different alleles of one gene is a common strategy during crop evolution and adaptation.

Genome-editing techniques have been developed to introduce precise, predictable genome modifications into plants in order to obtain the desired traits. These techniques are giving rise to precision breeding techniques that are defining the next-generation of plant breeding^24–26^. CRISPR/Cas9-mediated genome editing has been widely used to create novel allelic variation in plants^27,28^. For example, new alleles developed by gene editing of the MADS-box genes *J2*, *EJ2*, and *LIN* in tomato have allowed inflorescence architecture to be optimized, leading to improved yields^29^. In recent years, several cases of multiplex gene editing have drastically accelerated gene stacking for important traits. In a recent example, 13 rice pyrabactin resistance 1(PYR1)/PYR1-like (PYL) family genes were edited by CRISPR/Cas9. Characterization of the combinatorial mutants suggested that *pyl1/4/6* exhibited the best growth and improved grain productivity in natural paddy field conditions^30^. Here, we generated all combinatorial null mutants of four *LHY* homologs by CRISPR/Cas9 and found that *lhy1a/1b/2b* exhibited the most improved grain yield under natural SD conditions (Fig. 3), but *lhy1a* (*Tof16*) and *lhy1b/2a/2b* had the highest grain production under natural LD conditions (Supplementary Fig. 14).

Perhaps combining natural variation alleles of the four *LHY* homologs could also improve soybean productivity (Fig. 3h). In addition, combining loss-of-function alleles of *Tof16* and *J* could allow us to create higher-yielding soybean varieties for growth in tropical regions (Fig. 3i). Therefore, our findings not only shed light on the soybean adaptation trajectory into the tropics, but they also lay the foundation for improving soybean productivity.

## Methods

### Resequencing, mapping, and variation calling

The libraries for each accession of 329 panel were constructed following the manufacturer’s instructions (Illumina Inc., San Diego, CA, USA). The DNA-seq libraries were sequenced on the HiSeq X Ten system (150 bp Paired-end reads). Resequencing reads of the 329 accessions sequenced in this study and the 1295 previously sequenced accessions mapping and SNP calling were performed as described previously^14^. In brief, paired-end resequencing reads were mapped to the Williams 82 genome (Wm82.a2.v1)^17^ with BWA 0.6.1-r104 software with default parameters^31^. The SNPs and indels were called with GATK (ver. 3.1.1)^32,33^ and SAMtools (ver. 0.1.19) software^34^, independently; then, the common sites identified by both methods were retained for further analysis. SNPs with missing data minor allele frequency (MAF) <1% were filtered, and indels with a maximum length of 10 bp were included. Annotations of SNP and INDEL were performed based on the Williams 82 genome using snpEff (ver. 3.1) software^35^. SNPs in coding sequences were classified as synonymous SNPs or nonsynonymous SNPs. Indels in exons were further categorized according to whether they led to a frameshift.

### Population genetic analysis

To conduct phylogenetic analysis, SNPs with MAF < 0.05 were filtered out for all soybean accessions. The remaining SNPs were used to construct a neighbor-joining tree with MEGA v6.06 software and were visualized with the online tool iTOL (https://itol.embl.de). PCA was performed with this SNP set with the smartpca program in the EIGENSOFT v.5.0.1package^36,37^.

### Linkage disequilibrium analysis

Linkage disequilibrium was calculated for each subpopulation with SNPs with MAF > 0.05. To perform the linkage disequilibrium calculation, plink v1.9 software was applied with the parameters (–ld-window-r2 0–ld-window 99999–ld-window-kb 1000). Linkage disequilibrium decay was calculated based on *r*^*2*^ between two SNPs and averaged in 1-kb windows with a maximum distance of 1 Mb (ref. ^38^).

### GWAS for flowering time

We used 4,435,213 high-quality SNPs (MAF > 0.05) to perform GWAS for flowering time in the 329 accessions. Association analyses were performed by MLM implemented in efficient mixed-model association expedited (EMMAX) software^20^. Kinship was derived from all of these SNPs. The threshold for GWAS was determined by Bonferroni correction (that is, corrected *P* = 0.05/*n*, in which *n* is the number of independent SNPs across the genome). The significantly associated regions were manually verified from the aligned resequencing reads against the Williams genome with SAMtools v0.1.18 (ref. ^34^).

### Soybean accessions, growth conditions, and phenotyping

The 329-accession panel was grown during the cultivation season (July to December) in 2018 and 2019 at the experimental station of the Guangzhou University in Guangzhou (23° 16′N, 113°23′E). Flowering time was investigated in 2018 and 2019. For map-based cloning, one heterozygous inbred progeny population of 2418 individuals segregating at the *Tof16* locus was subsequently developed. NILs for the *Tof16* locus were selected from F7 progeny of this same cross using molecular markers for *Tof16*. The heterozygous inbred progenies, NILs, and CRISPR–Cas9 knockout mutants used for phenotyping were grown under natural SD conditions in the field (day length 13 h light/11 h dark) from 2018 to 2020 at the Experimental Station of the Guangzhou University or under natural LD conditions (day length 15 h light/9 h dark) in 2020 at the Experimental Station of the Jilin Academy of Agricultural Sciences. For natural SD conditions, plants were sown in the beginning of July, spaced 0.15 m apart in rows 5 m long, with 0.7 m between rows, and harvested in November or December of each year. For natural LD conditions, plants were sown in the beginning of May, spaced 0.15 m apart in rows 5 m long, with 0.7 m between rows, and harvested in September or October. Plants used for expression analysis and ChIP assays were grown under SD conditions (day length 12 h light/12 h dark) in a plant growth cabinet (Conviron Adaptis A1000) with a light intensity of 500 μmol m^−2^ s^−1^.

For phenotypic investigations, the days from emergence to the first flowering, corresponding to the R1 stage^39^ were scored. The days from emergence to pods attained mature color, corresponding to the R8 stage^39^ were scored. Yield-related traits were recorded at the R8 stage^12^.

### DNA isolation and map-based cloning

Genomic DNA was extracted from fresh trifoliate leaves of 2-week-old seedlings with a SurePlant DNA kit (CWBIO) and used to amplify indel markers. The primer sequences used to amplify the markers for mapping are listed in Supplementary Table 9. For fine mapping, dCASP markers were developed in the regions of *Tof16* based on the resequencing data of the two parents, PI591429 and PI628930. Seven recombinants were identified in the fine-mapping population of *Tof16* using seven markers. The flowering time of the progeny of these recombinants was evaluated to delimit the genomic interval containing *Tof16*.

### RNA extraction and quantitative RT-PCR

Total RNA was extracted using an Ultrapure RNA kit (CWBIO) and the RNA was reversely transcribed using a Super Script First-strand cDNA Synthesis System (Takara, Dalian, China). Quantitative reverse-transcription PCR (qRT-PCR) was performed using SYBR Green Real-Time PCR Master Mix (Roche). Three independent RNA samples were prepared for biological replicates. The soybean *Tubulin* (*Glyma.05G157300*) gene was used as the internal reference. All qPCR primers are listed in Supplementary Table 5.

### Plasmid construction and plant transformation

The coding sequence of *Tof16* was amplified with the primer set Tof16-6HA-F/R and inserted into the *Xba* I and *Mlu* I sites of the pTF101-6HA vector (containing the *Bar* gene for glufosinate resistance) to generate the *pro35S*-*Tof16*-*6HA* construct. The recombinant vector was introduced into *Agrobacterium tumefaciens* strain *EHA101* and used to transform Williams 82 via *Agrobacterium tumefaciens*-mediated transformation^40,41^.

### Transient expression assay

To generate the *proE1-LUC* reporter construct, the 3147-bp promoter sequence of *E1* was amplified from Williams 82 and introduced into the pGreenII 0800-LUC vector^42^. The different alleles of *Tof16* (*Tof16*, *tof16-1*, *tof16-2*, *tof16-3*, *tof16-4*) were introduced into the *pTF101-6HA* vector to generate the constructs *pro35S-Tof16-6HA*, *pro35S-Tof16-1-6HA*, *pro35S-Tof16-2-6HA*, *pro35S-Tof16-3-6HA*, and *pro35S-Tof16-4-6HA*. The different alleles of *J* (*J-H1*, *J-H28*, *j-11*) were introduced into the *pTF101-6HA* vector to generate the constructs *pro35S-J-H1-6HA*, *pro35S-J-H1-6HA*, and *pro35S-j-11 -6HA*. The *proE1-LUC* construct was used as the reporter and various *Tof16* and *J* constructs were used as the effectors in the *Arabidopsis* protoplast transient expression system to test whether Tof16 and J suppress the transcription of *E1*.

### Immunoblot analysis

To analyze protein expression in the transgenic plants, total proteins were extracted from Williams 82 and the *p35S-Tof16-6HA* transgenic lines in protein extraction buffer (50 mM Tris–HCl pH 7.5, 150 mM NaCl, 5 mM EDTA, 0.1% Triton X-100 and protease inhibitor cocktail) and used for immunoblot analysis. Immunoblot analysis was performed as described previously^12^. In brief, total proteins were separated by SDS-PAGE. After electrophoresis, the proteins were transferred to polyvinylidene difluoride membranes (Millipore) and probed using antibodies anti-HA antibody (ab18181, 1:5000 dilution). The anti-HA antibody (ab18181) was obtained from Abcam.

### ChIP assay

Leaf samples were collected from 20-day-old seedlings at Zeitgeber time 0 under SD conditions from Williams 82 and *p35S-Tof16-6HA* transgenic lines. The samples were fixed in 1% formaldehyde on ice for 15 min under a vacuum. Nuclei were isolated from the samples and sonicated to generate DNA fragments with an average size of ~250–500 bp. The solubilized chromatin was immunoprecipitated by Protein G PLUS agarose (16-201, Millipore) with anti-HA antibody (ab18181). The coimmunoprecipitated DNA was recovered and analyzed by qRT-PCR in triplicate. Data normalized with input transcripts are means from three biological repeats. The enrichment of the *EF1b* genomic fragment was used as a negative control. The primers used for amplification are listed in Supplementary Table 5.

### Statistical analyses

In this study, all values were presented as mean ± s.e.m. and numbers (*n*) of samples or replicates are indicated in figure legends. Data were analyzed with GraphPad Prism 8 (ver. 8.0.1). Significance levels of differences were calculated by one-tailed, two-sample Student’s *t*-tests or one-way ANOVA with GraphPad Prism 8 (ver. 8.0.1). For 329 accessions phenotypic evaluation, at least 10 individual plants were analyzed.

### Characterization of diversity panel

Resequencing of the newly assembled 329-accession diversity panel (including 110 wild soybeans, 45 landraces, and 174 improved cultivars) generated a total of 21.8 billion paired-end reads of 100 bp in length (6.5 Tb of sequences), with an average coverage depth of more than 10x for each line. After mapping to the reference genome of soybean cultivar Williams 82 (W82)^17^, 31,925,948 SNPs and 3,266,897 indels (shorter than or equal to 6 bp) were identified (Supplementary Table 6). In addition, the depth of sequencing data allowed us to identify a total of 704 tandem duplications, 7160 segmental deletions, 3781 segmental insertions, and 497 inversions (Supplementary Table 6).

### Genome-wide association analyses

To identify loci influencing flowering time in the 329-accession panel, we conducted a GWAS through the MLM implemented in Efficient Mixed-Model Association eXpedited (EMMAX) software^20^ based on the SNPs with a MAF > 0.05 and using the flowering time at GuangZhou in 2018 and 2019 for each accession.

### Reporting summary

Further information on research design is available in the Nature Research Reporting Summary linked to this article.

## Supplementary information

Supplementary Information file

Supplementary Data 1

Supplementary Data 2

Supplementary Data 3

Supplementary Data 4

Description of Additional Supplementary Files

Reporting Summary

## Data Availability

The data supporting the findings of this work are available within the paper and its Supplementary Information files. A reporting summary for this article is available as a Supplementary Information file. The sequencing data (329 accessions) used in this study have been deposited into the NCBI database under accession number PRJNA728982. The previously reported sequence data^14^ were deposited into the NCBI database under accession number PRJNA394629 and the GSA database in BIG Data Center under accession number PRJCA000205 and PRJCA001691. Source data are provided with this paper.

## Source data

Source data
