# Supplementary material for: Genetic basis and adaptation trajectory of soybean from its temperate origin to tropics
Source: Nat Commun. 2021 Sep 14;12:5445. doi: 10.1038/s41467-021-25800-3 (PMC8440769; doi:10.1038/s41467-021-25800-3)
Supplement: Supplementary file 1 — Supplementary Information file [file 41467_2021_25800_MOESM1_ESM.pdf]

Genetic basis and adaptation trajectory of soybean from its temperate origin to tropics

Dong *et al.*

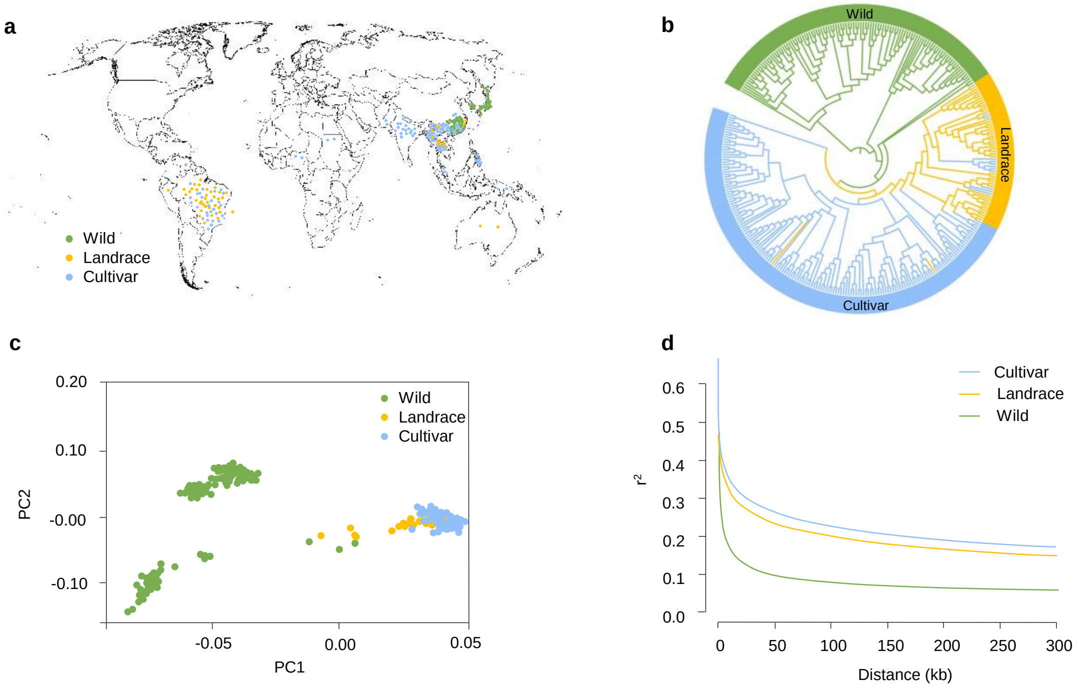

Supplementary Fig. 1 Characterization of soybean diversity panels.
a Geographic origins of a newly assembled 329-accession diversity panel. The map was drawn using ArcGIS v.10.3 software for desktop (https://desktop.arcgis.com/en/). **b** Phylogenetic structure of the diversity panel based on analysis of genome-wide SNPs. The apparent misclassification reflects an identification error in the passport data. **c** PCA of genetic diversity within the panel. **d** Linkage disequilibrium decay for the three subgroups within the diversity panel. Linkage disequilibrium decay is determined by squared correlations of allele frequencies (*r*2) against the distance between polymorphic sites in wild soybeans (gray), landraces (green) and cultivars (blue).

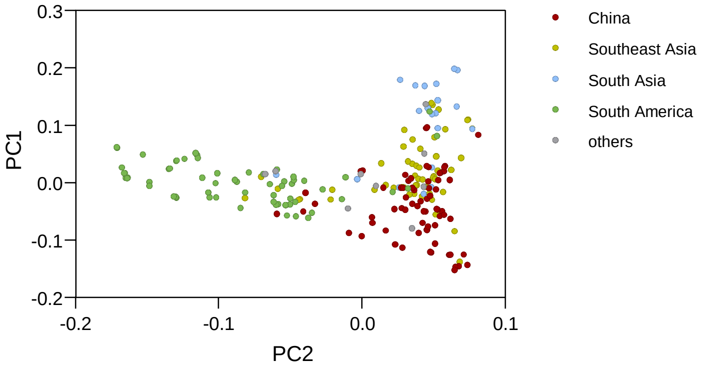

Supplementary Fig. 2 PCA with only the cultivars and color them by country of origin.

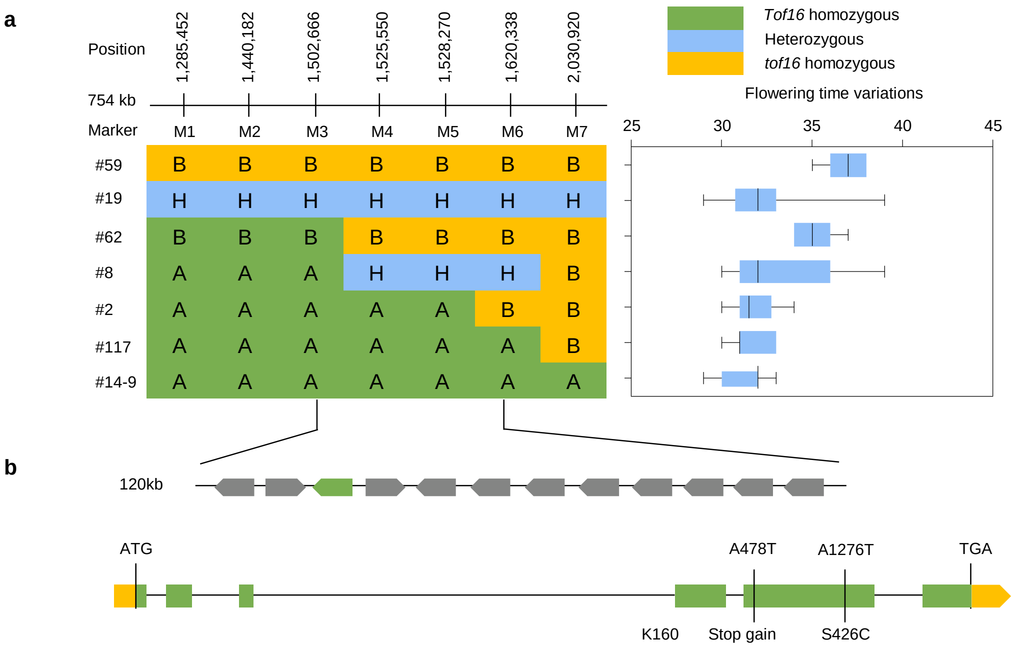

Supplementary Fig. 3 Positional cloning of *Tof16*.
a Characterization of key recombinants in the immediate vicinity of the Tof16 locus showed recombination break points (left panel), and mean flowering time of progeny (right panel). (n = 40 plants), The lower and upper box edges corresponded to the first and third quartiles (the twenty-fifth and seventy-fifth percentiles); the horizontal line indicated the median value; and the lower and upper whiskers corresponded to the smallest value at most 1.5× interquartile range and the largest value no further than 1.5× interquartile range. **b** Gene structure of *Tof16* showed the location of the loss-of-function *tof16-1* mutation. Source data are provided as a Source Data file.

```
Tof16      MDAYSSGEEVVVKTRKPYTITKQRERWTEEEHNRFLEALKLHGRAWQRIEEHIGTKTAVQIRSHAQKFFTKLEKEALVKGVPIGQALDIDIPPPRPKRKPNNPYPRKTRIGTTSLHSGAK   120
tof16-1    MDAYSSGEEVVVKTRKPYTITKQRERWTEEEHNRFLEALKLHGRAWQRIEEHIGTKTAVQIRSHAQKFFTKLEKEALVKGVPIGQALDIDIPPPRPKRKPNNPYPRKTRIGTTSLHSGAK   120
tof16-2    MDAYSSGEEVVVKTRKPYTITKQRERWTEEEHNRFLEALKLHGRAWQRIEEHIGTKTAVQIRSHAQKFFTKLEKEALVKGVPIGQALDIDIPPPRPKRKPNNPYPRKTRIGTTSLHSGAK   120

Tof16      DGKLNLVESSHVNQALDLKKEPLPEKHDLDEGLTTVKENKDENHAKVFTLLQEVPCSSVSSANESSITMSVPLGNPCAFKEITPSVKEVIARDEKTESFVTVEPENGKLEINDGKQTNGT   240
tof16-1    DGKLNLVESSHVNQALDLKKEPLPEKHDLDEGLTTVKEN.................................................................................   159
tof16-2    DGKLNLVESSHVNQALDLKKEPLPEKHDLDEGLTTVKENKDENHAKVFTLLQEVPCSSVSSANESSITMSVPLGNPCAFKEITPSVKEVIARDEKTESFVTVEPENGKLEINDGKQTNGT   240

Tof16      SKDSRLEDSDALHMKLVQNEKPDGLDCELTIDGMQGNQNYPRHVTVHVVDGNLGTNTQNPSQDMLFRDSMFQPIGGVNGQRNVFTNTAPSNTSESQNNTARSSVHQSFLPYPPFTQHNQD   360
tof16-1    ........................................................................................................................   159
tof16-2    SKDSRLEDSDALHMKLVQNEKPDGLDCELTIDGMQGNQNYPRHVTVHVVDGNLGTNTQNPSQDMLFRDSMFQPIGGVNGQRNVFTNTAPSNTSESQNNTARSSVHQSFLPYPPFTQHNQD   360

Tof16      DYQSFLHMSSTFSNLIVSTLMQNPAAHAAASFAATFWPYANPETSANSPRCSQGGFTNRQIGSPPSVAAIAAATVAAATAWWAAHGLLPLCAPLHTSFACPASVTTVPSMNTGEAPALKA   480
tof16-1    ........................................................................................................................   159
tof16-2    DYQSFLHMSSTFSNLIVSTLMQNPAAHAAASFAATFWPYANPETSANSPRCSQGGFTNRQIGSPPCVAAIAAATVAAATAWWAAHGLLPLCAPLHTSFACPASVTTVPSMNTGEAPALKA   480

Tof16      EQEKTTLQNPPLQDQMLDPEYSEAQQAQHSASKSPAAILSDSESGDAKLNTSSKVTDHETNKTISEHLDSNKTKGRKPVDRSSCGSNTASSSDVETDALEKGEKGKEEPEIPDANQLAIE   600
tof16-1    ........................................................................................................................   159
tof16-2    EQEKTTLQNPPLQDQMLDPEYSEAQQAQHSASKSPAAILSDSESGDAKLNTSSKVTDHETNKTISEHLDSNKTKGRKPVDRSSCGSNTASSSDVETDALEKGEKGKEEPEIPDANQLAIE   600

Tof16      FSNRRRSVSNLTDSWKEVSEEGRLAFQALFSREVLPQSFSPPHALKNTDHQMDNANDNKQNIDDKDEDLDGSKKCSSNYEAMQKNLLFVENNEGLLTIGLGQGKLKTHRTGFKPYKRCSM   720
tof16-1    ........................................................................................................................   159
tof16-2    FSNRRRSVSNLTDSWKEVSEEGRLAFQALFSREVLPQSFSPPHALKNTDHQMDNANDNKQNIDDKDEDLDG.KKCSSNYEAMQKNLLFVENNEGLLTIGLGQGKLKTHRTGFKPYKRCSM   719

Tof16      EAKENRVGASSNQGEEQGCKRIRLEGETS                                                                                              749
tof16-1    .............................                                                                                              159
tof16-2    EAKENRVGASSNQGEEQGCKRIRLEGETS                                                                                              748
```

Supplementary Fig. 4 Protein sequence comparisons of *Tof16* alleles.
Red frame indicates the Myb domain of LHY1a. The black arrows represent the variation of *tof16-2* .

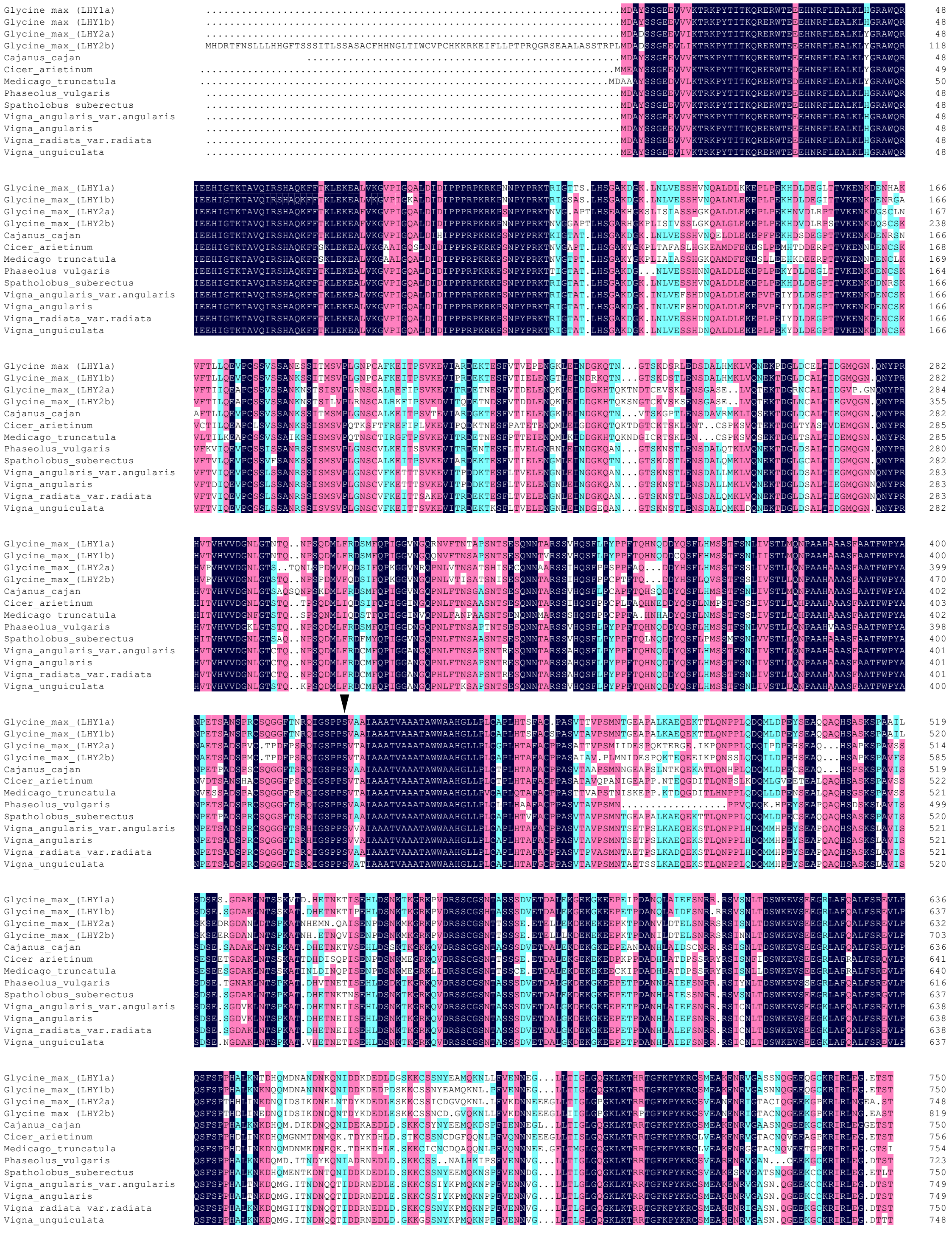

Supplementary Fig. 5 Comparison of the amino acid sequences of Tof16 and its homologues in legumes.
The black arrow represent the variation of *tof16-2*.

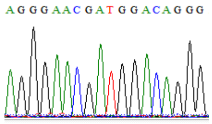

Tof16	**AGGAACGATGGACAGAGG**
tof16^{CR}	**AGGAACGATGGACAG-GG**

Supplementary Fig. 6 CRISPR/Cas9-induced 1bp deletion of *Tof16.*

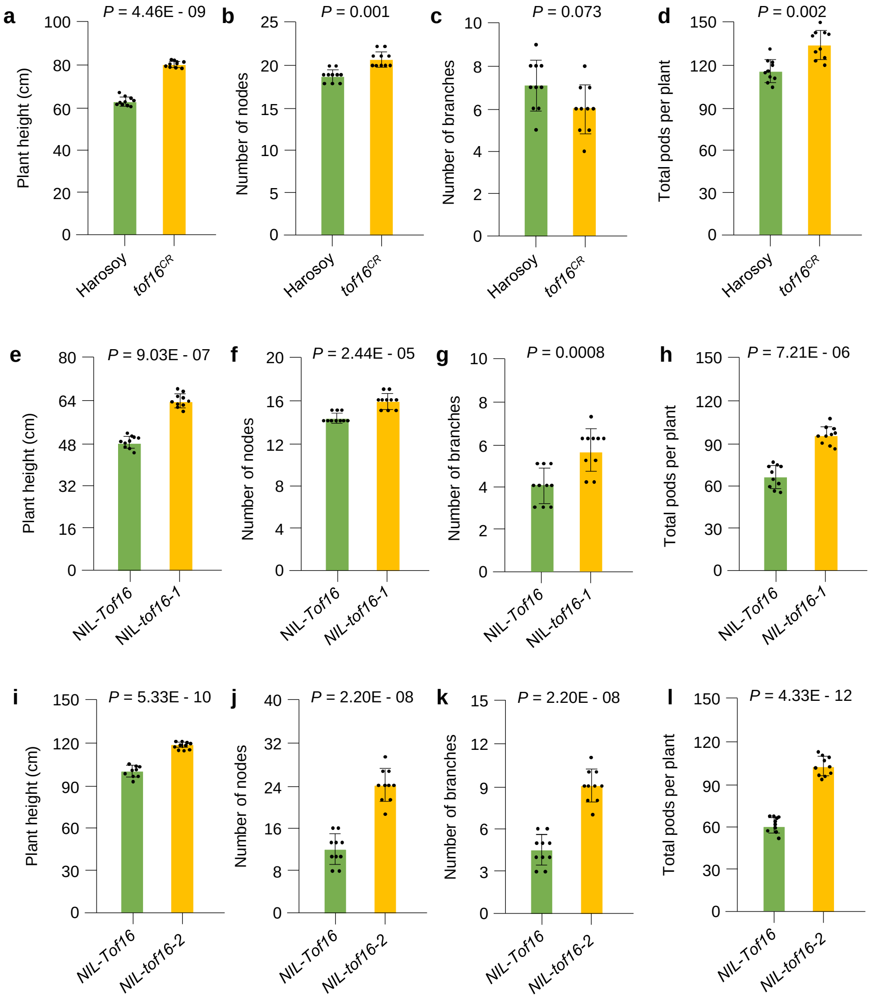

Supplementary Fig. 7 Phenotypes of NILs of *Tof16*, Harosoy and *tof16*^{CR} under SD (12 h light/12 h dark) conditions. **a** Plant height. **b** Number of nodes, **c** Number of branches. **d** Total pods per plant of Harosoy and *tof16*^{CR}. **e** Plant height. **f** Number of nodes. **g** Number of branches. **h** Total pods per plant of NIL-*Tof16* and NIL-*tof16-1*. **i** Plant height. **j** Number of nodes. **k** Number of branches. **l** Total pods per plant of NIL-*Tof16* and NIL-*tof16-2*. All data were given as mean ± s.e.m. (n = 10 plants), the value of each plant was represented by a dot. One-tailed Student's t-test was used to generate the P values. Source data are provided as a Source Data file.

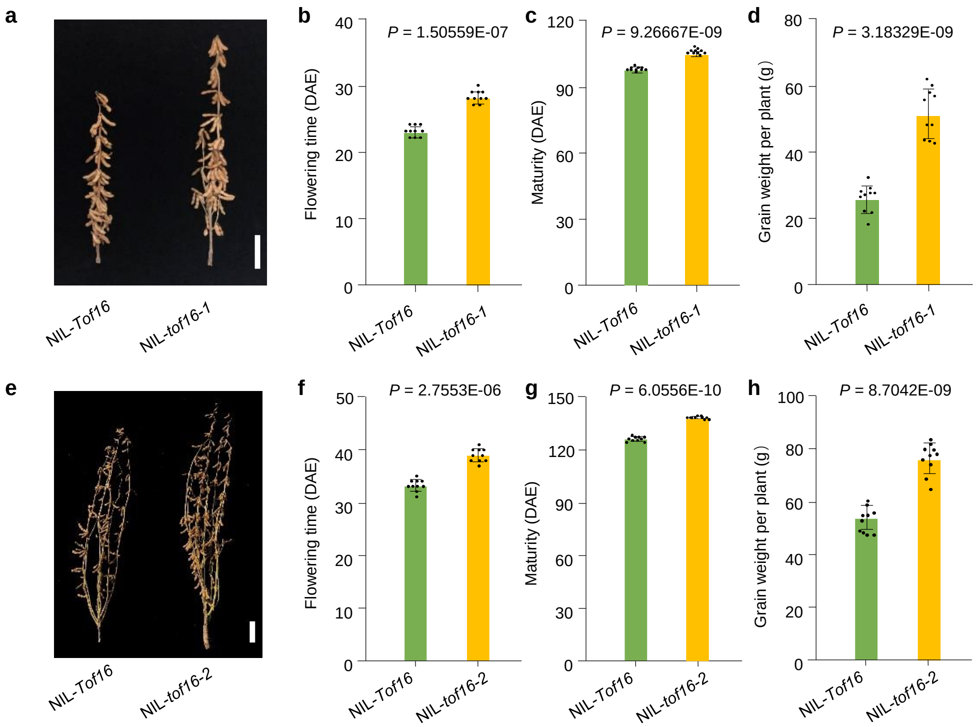

Supplementary Fig. 8 Phenotypes of NILs of *Tof16* under SD (12 h light/12 h dark) conditions.
a Phenotypes of NIL-*Tof16* and NIL-*tof16-1*. Scale bar, 10 cm. **b** Flowering time. **c** Time to maturity. **d** Grain yield per plant of NIL-*Tof16* and NIL-*tof16-1*. **e** Phenotypes of NIL-*Tof16* and NIL-*tof16-2*. Scale bar, 10 cm. **f** Flowering time. **g** Time to maturity. **h** Grain yield per plant of NIL-*Tof16* and NIL-*tof16-2*. All data were given as mean ± s.e.m. (n = 10 plants), the value of each plant was represented by a dot. One-tailed Student's t-test was used to generate the P values. Source data are provided as a Source Data file.

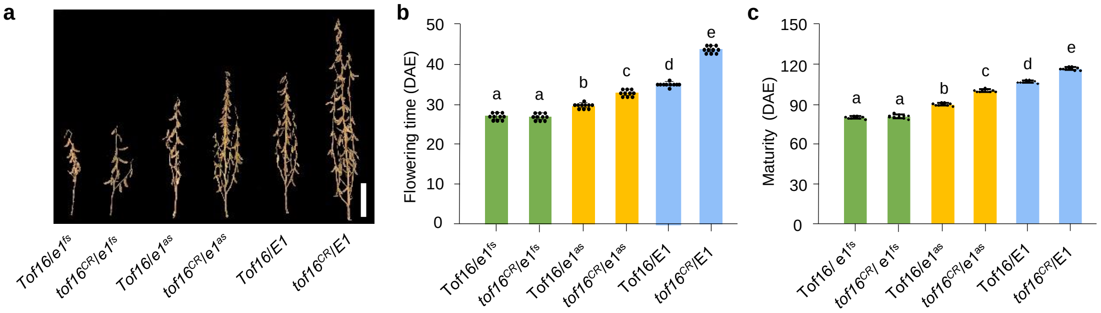

Supplementary Fig. 9 Genetic relationship of *Tof16* and *E1.*
a Phenotypes of NILs possessing different allelic combinations at *Tof16* and *E1* under SD (12 h light/12 h dark) conditions. $e1^{as}$, partially functional *e1* allele. $e1^{fs}$, *e1* null functional allele. Scale bar, 10 cm. **b** Flowering time. **c** Time to maturity of NILs possessing different allelic combinations at *Tof16* and *E1*. All data were given as mean ± s.e.m. (n = 10 plants), the value of each plant was represented by a dot. The presence of the same lowercase letter above the histogram bars in b, c denoted nonsignificant differences across the two panels ($P > 0.05$). One-way ANOVA was used to generate the *P* values. Source data are provided as a Source Data file.

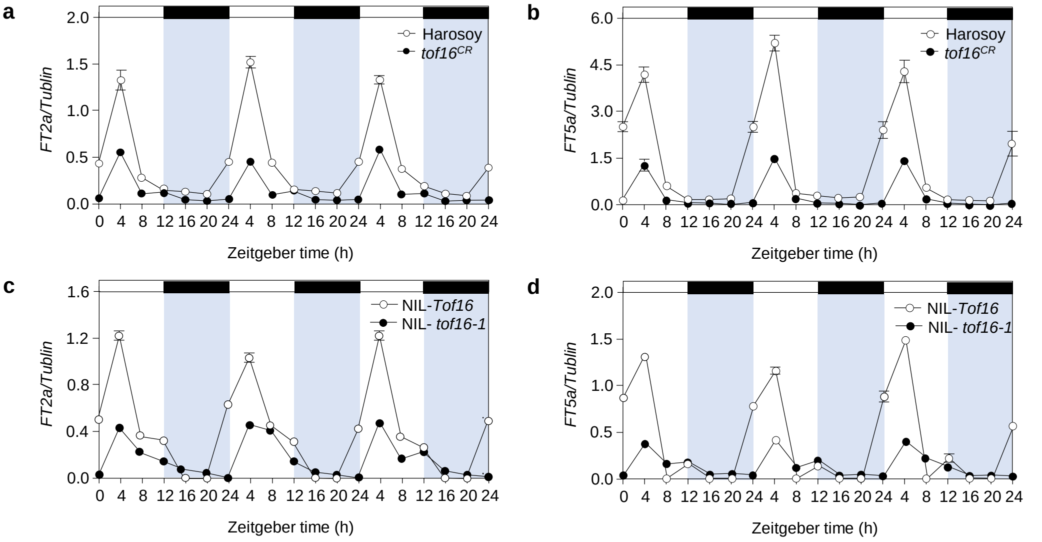

Supplementary Fig. 10 Expressions of *FT2a* and *FT5a* in NIL-*Tof16* and NIL- *tof16-1*, and Harosoy and *tof16*^{CR} mutant.
a-b Expressions of *FT2a* (**a**) and *FT5a* (**b**) in Harosoy and *tof16*^{CR} mutant. **c-d** Expressions of *FT2a* (**c**) and *FT5a* (**d**) in *NIL-Tof16 and NIL-tof16*. All data are given as mean ± s.e.m. (n = 5 plants). Plants were grown under SD (12 h light/12 h dark) and sampled at 20 DAE. Source data are provided as a Source Data file.

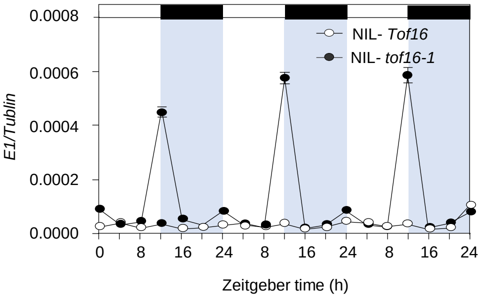

Supplementary Fig. 11 Expressions of *E1* in NIL-*Tof16* and NIL- *tof16-1*.
All data are given as mean ± s.e.m. (n = 5 plants). Plants were grown under SD (12 h light/12 h dark) and sampled at 20 DAE. Source data are provided as a Source Data file.

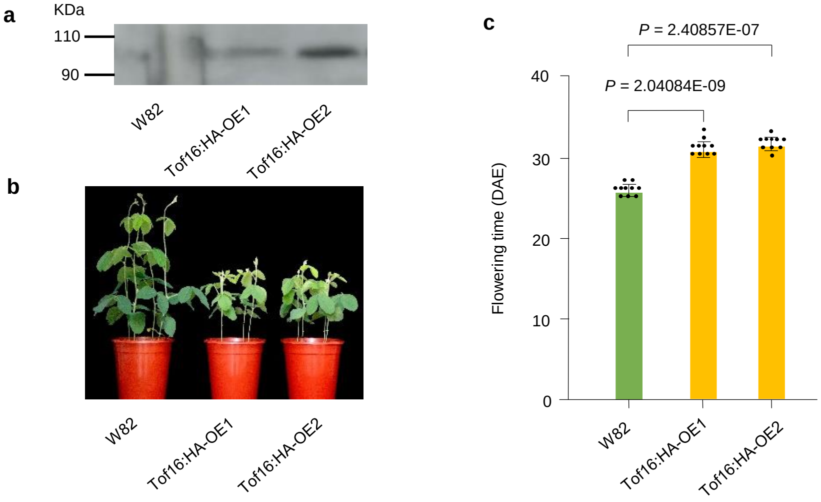

Supplementary Fig. 12 Overexpression of *Tof116-HA* in soybean cultivar Williams 82 (W82).
a Imuno blot of HA antibodies in two independent *Tof12-HA* over expression (OE) lines and W82. The original gel blot images see the source data in Supplementary Fig. 26. **b** Phenotypes of two independent OE lines and W82. **c** Flowering time of two OE lines and W82 under SD (12 h light/12 h dark) conditions. All data are given as mean ± s.e.m. (n = 10 plants). One-tailed Student's *t*-test was used to generate the *P* values. Source data are provided as a Source Data file.

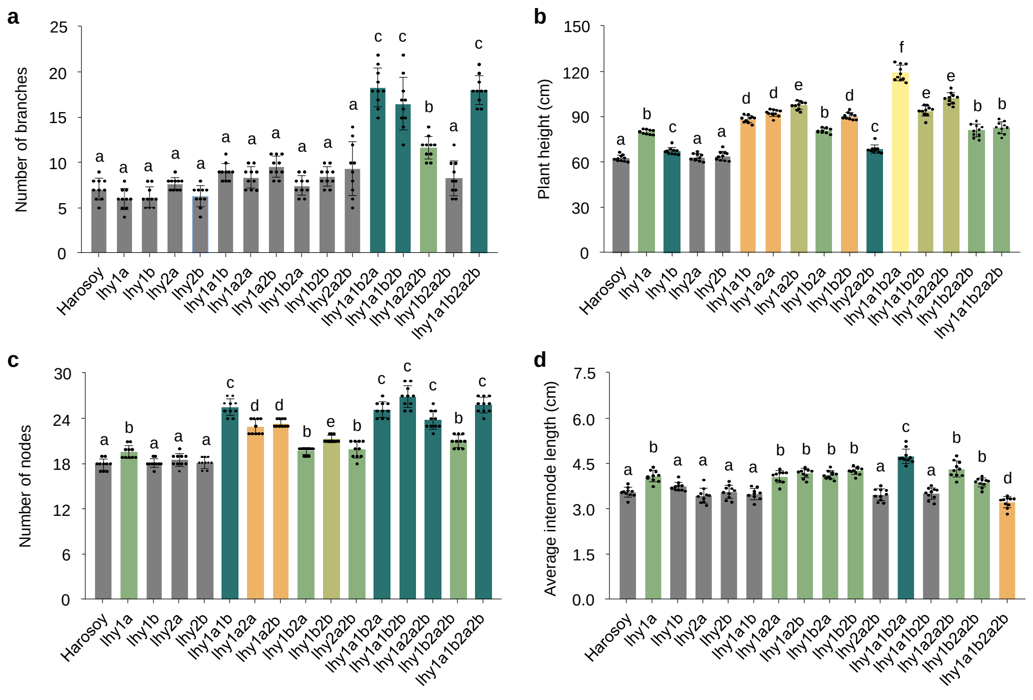

Supplementary Fig. 13 Phenotypes of *lhy* mutants under short-day.
a Number of branches. **b** Plant height. **c** Number of nodes. **d** Average internode length. All data were given as mean ± s.e.m. (*n* = 10 plants), the value of each plant was represented by a dot. The presence of the same lowercase letter above the histogram bars in **a**, **b**, **c** ,**d** denotes nonsignificant differences across the two panels (*P* > 0.05). One-way ANOVA was used to generate the *P* values. Source data are provided as a Source Data file.

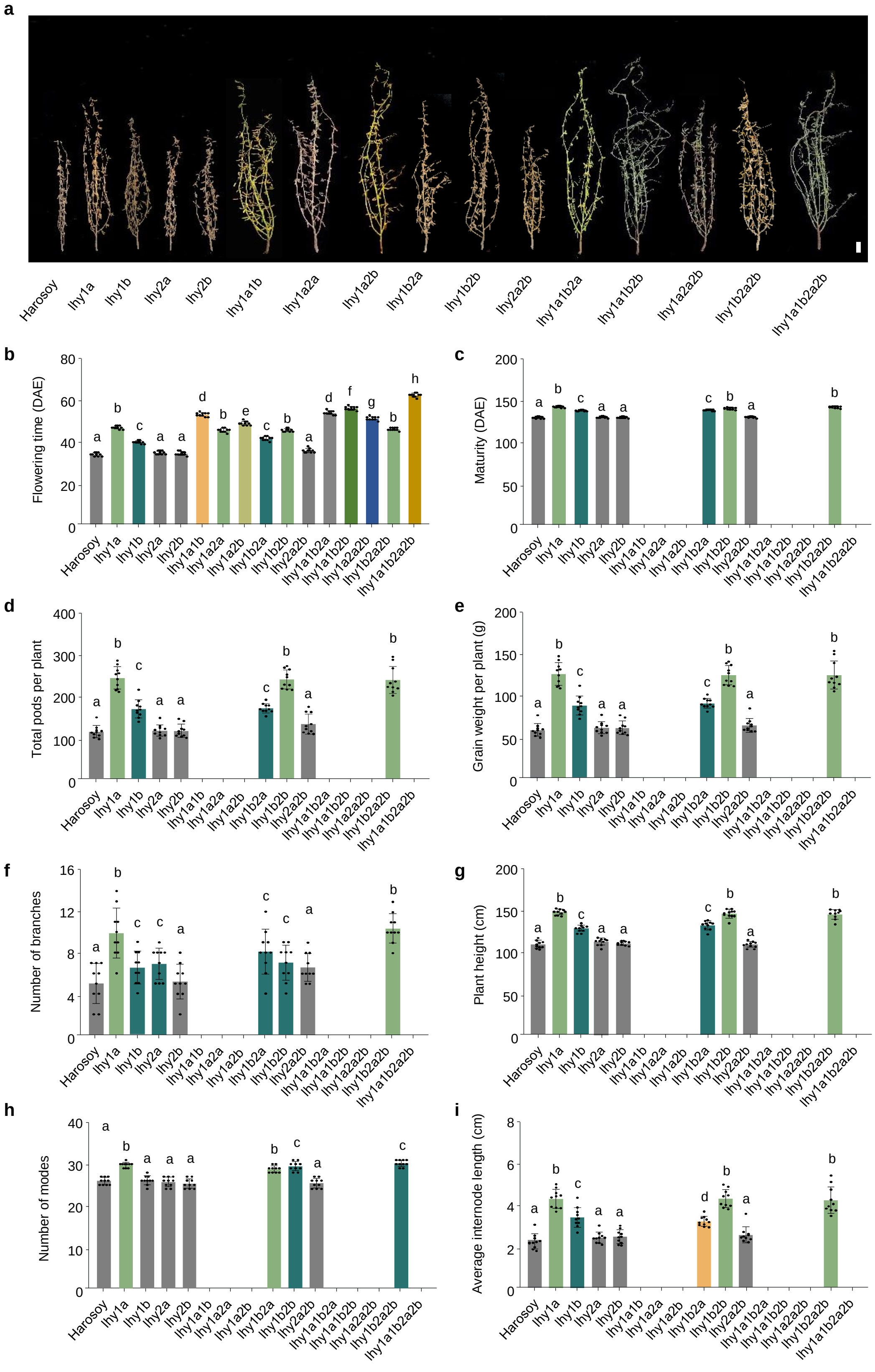

Supplementary Fig. 14 Redundancy among four *LHY* genes regulates soybean flowering time and yield under long-day.
a Phenotypes of *lhy* mutants. Scale bar, 10 cm. **b** Flowering time. **c** Time to maturity. **d** Total pods per plant height. **e** grain yield per plant. **f** Number of branches. **g** Plant height. **h** Number of nodes. **i** Average internode length. All data were given as mean ± s.e.m. (n = 10 plants), the value of each plant was represented by a dot. The presence of the same lowercase letter above the histogram bars in **b**, **c**, **d**, **e**, **f**, **g**, **h**, **i** denotes nonsignificant differences across the two panels (P > 0.05). One-way ANOVA was used to generate the P values. Source data are provided as a Source Data file.

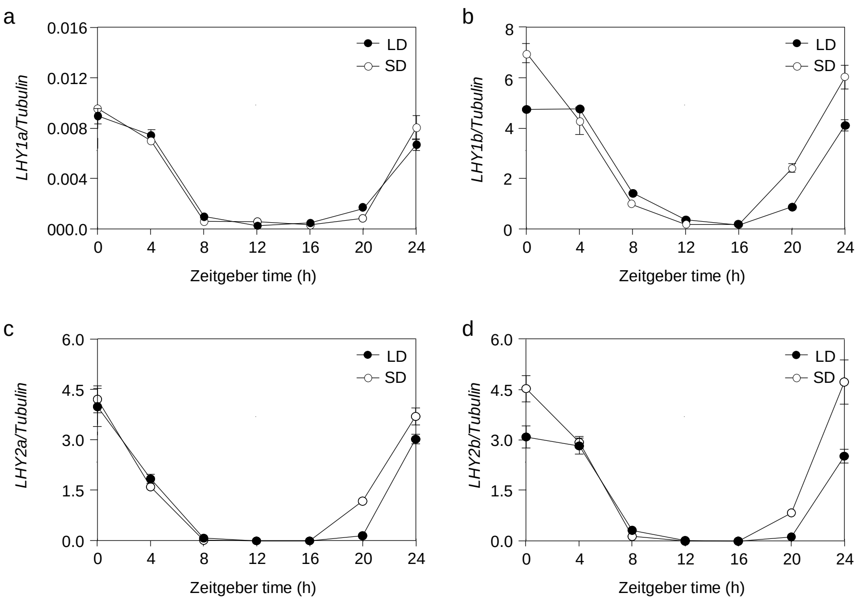

Supplementary Fig. 15 Expressions of *LHY1a*, *LHY1b*, *LHY2a and LHY2b* in Harosoy under LD and SD conditions.
All data are given as mean ± s.e.m. (n = 5 plants). Plants were grown under LD (16 h light/8 h dark) and SD (12 h light/12 h dark) and sampled at 20 DAE. Source data are provided as a Source Data file.

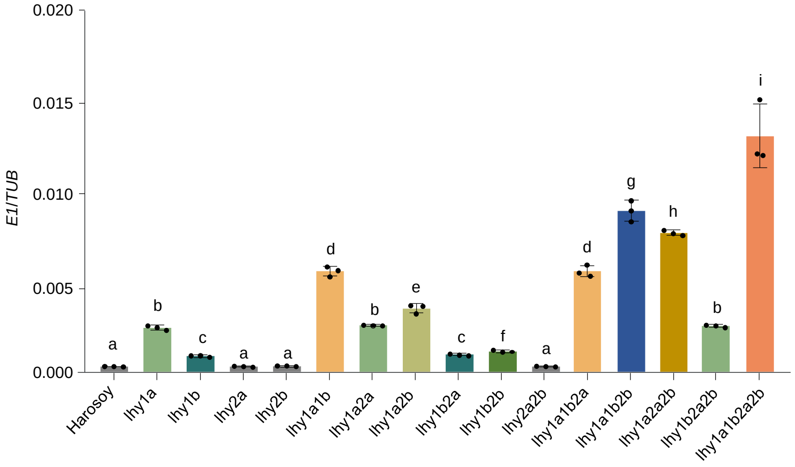

Supplementary Fig. 16. Expressions of *E1* in Harosoy and *lhy* mutants under SD conditions.
All data are given as mean ± s.e.m. (n = 5 plants). The presence of the same lowercase letter above the histogram bars denotes nonsignificant differences across the two panels ($P > 0.05$). Plants were grown under SD (12 h light/12 h dark) and sampled at 20 DAE (ZT12). Source data are provided as a Source Data file.

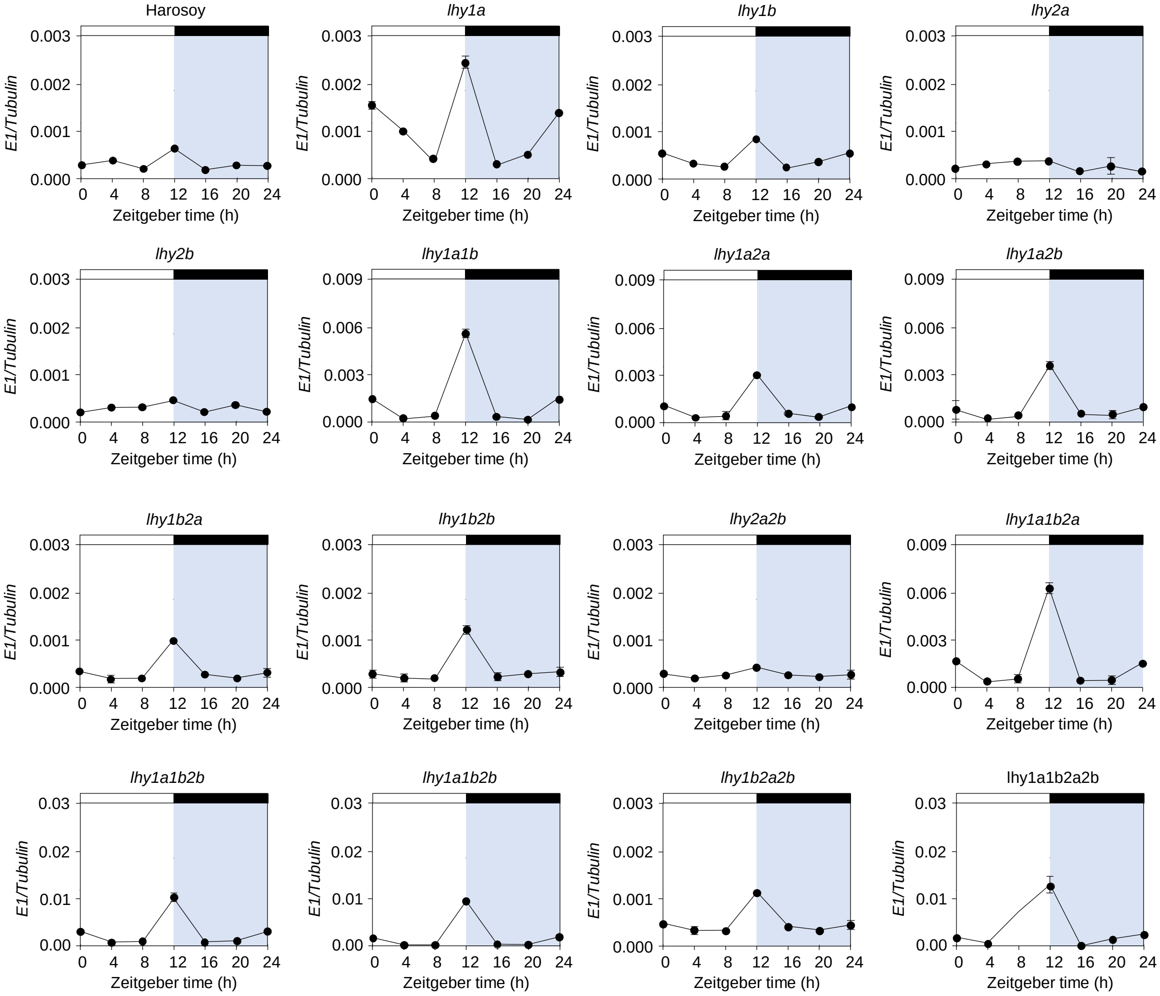

Supplementary Fig. 17 Diurnal expressions of *E1* in Harosoy and *lhy* mutants under SD conditions.
All data are given as mean ± s.e.m. (n = 5 plants). Plants were grown under SD (12 h light/ 12 h dark) and sampled at 20 DAE. Source data are provided as a Source Data file.

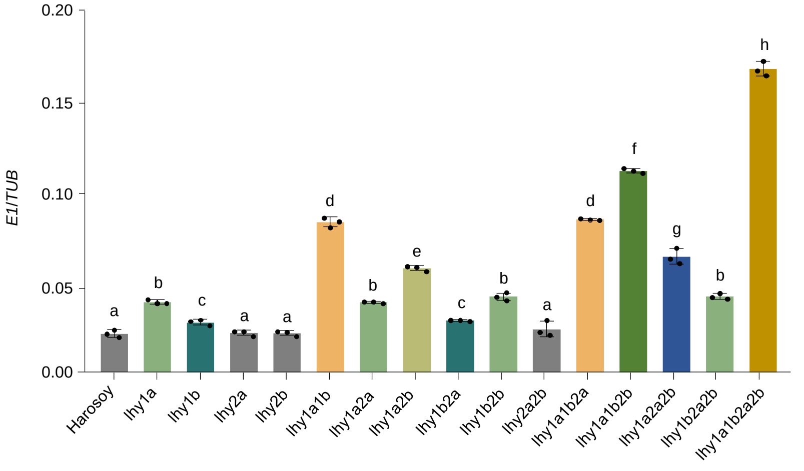

Supplementary Fig. 18 Expressions of *E1* in Harosoy and *lhy* mutants under LD conditions.
All data are given as mean ± s.e.m. (n = 5 plants). The presence of the same lowercase letter above the histogram bars denotes nonsignificant differences across the two panels ($P > 0.05$). Plants were grown under LD (16 h light/8 h dark) and sampled at 20 DAE (ZT16). Source data are provided as a Source Data file.

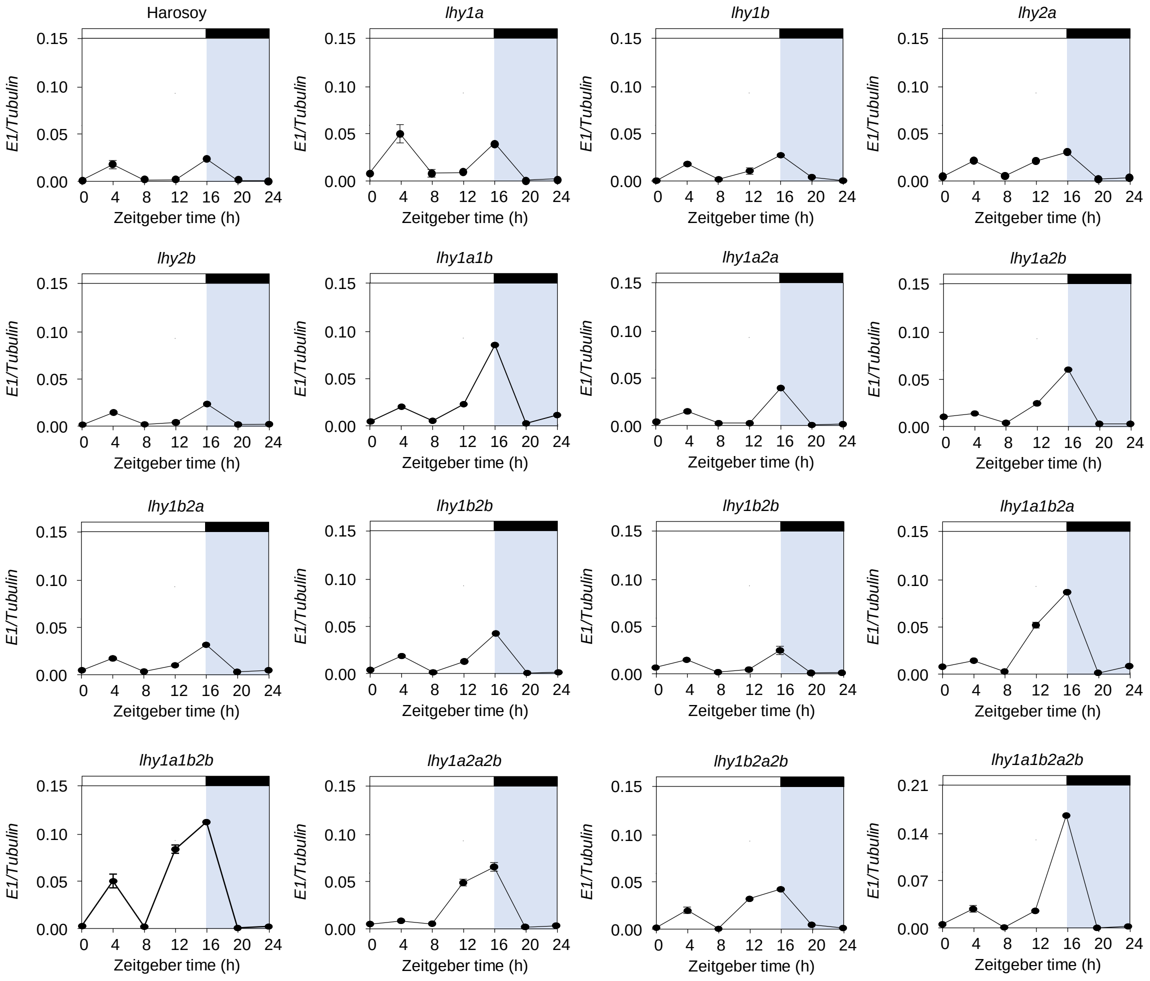

Supplementary Fig. 19 Diurnal expressions of *E1* in Harosoy and *lhy* mutants under LD conditions.
All data are given as mean ± s.e.m. (n = 5 plants). Plants were grown under SD (16 h light/ 8 h dark) and sampled at 20 DAE. Source data are provided as a Source Data file.

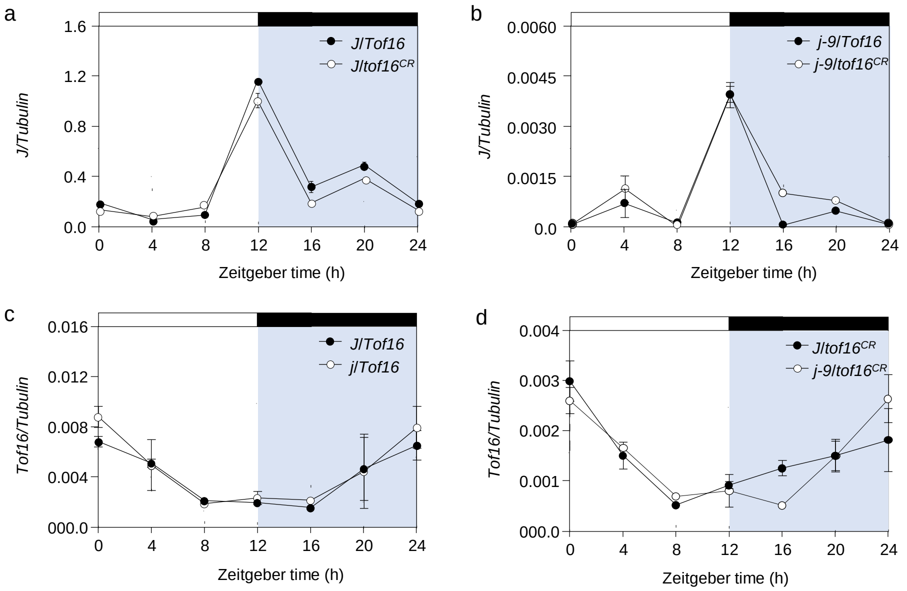

Supplementary Fig. 20 Expressions of *J* and *Tof16* in *J/Tof16*, *J/tof16^{CR}*, *j/Tof16* and *j/tof16^{CR}* mutants.
a Expressions of *J* in *J/Tof16* and *J/tof16^{CR}*. **b** Expressions of *J* in *j/Tof16* and *j/tof16^{CR}*. **c** Expressions of *Tof16* in *J/Tof16* and *J/tof16^{CR}*. **d** Expressions of *Tof16* in *j/Tof16* and *j/tof16^{CR}*. All data are given as mean ± s.e.m. (n = 5 plants). Plants were grown under SD (12 h light/12 h dark) and sampled at 20 DAE. Source data are provided as a Source Data file.

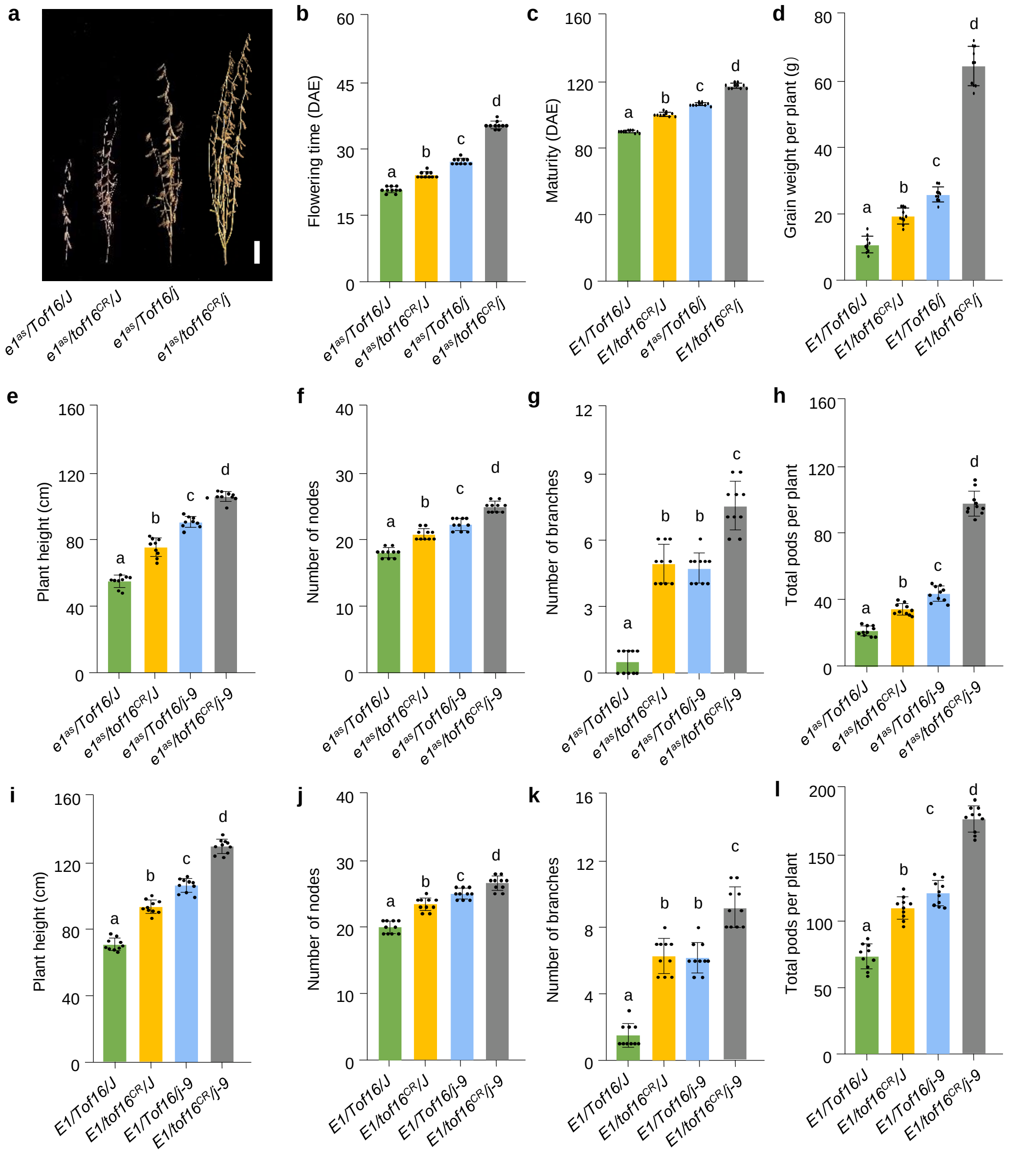

Supplementary Fig. 21 Phenotypes of NILs possessing different allelic combinations at *Tof16* and *J* under SD (12 h light/12 h dark) conditions. **a** phenotypes of NILs possessing different allelic combinations at *Tof16* and *J* in $e1^{as}$ background under SD (12 h light/12 h dark) conditions. $e1^{as}$, partially functional *e1* allele, Scale bar, 10 cm. **b** Flowering time, **c** time to maturity and **d** grain weight per plant. **e** Plant height, **f** Number of nodes, **g** Number of branches and **h** Total pods per plant of NILs possessing different allelic combinations at *Tof16* and *J* in $e1^{as}$ background . $e1^{as}$, partially functional *e1* allele. **i** Plant height, **j** Number of nodes, **k** Number of branches and **l** Total pods per plant of NILs possessing different allelic combinations at *Tof16* and *J* in *E1* background. All data were given as mean ± s.e.m. (n = 10 plants), the value of each plant was represented by a dot. The presence of the same lowercase letter above the histogram bars in **b**, **c**, **d**, **e**, **f**, **g**, **k**, **l** denoted nonsignificant differences across the two panels ($P > 0.05$). One-way ANOVA was used to generate the *P* values. Source data are provided as a Source Data file.

a

Haplotype	SNP Pos. / AA Change (Tof16)	338<br>T113I	442_444 del<br>148_148	461<br>T154R	478<br>K160X	562_630 del<br>188_210	821<br>M274K	822_823 ins<br>Q275fs	994<br>T332S	1102<br>M368L	1259<br>Q420L	1276<br>S426C	1864-downstream 510 del<br>F622-	1882<br>A628S	1927<br>H643Y	2015_2017 del<br>672_673	Number
H1																-	717
H2		T							T							-	5
H3		T								T						-	2
H4		T														-	12
H5			-													-	4
H6				G												-	3
H7							A									-	1
H8	*tof16-3*							TG								-	3
H9											T					-	3
H10	*tof16-1*				T							T				-	6
H11	*tof16-2*											T				-	496
H12	*tof16-4*											T	-	-	-	-	15
H13														T		-	1
H14															T	-	6
H15																	157
REF.W82		C	GAT	C	A	ACA....ATA	T	C	A	A	A	A	GGG...TCC	G	C	GCA	

b

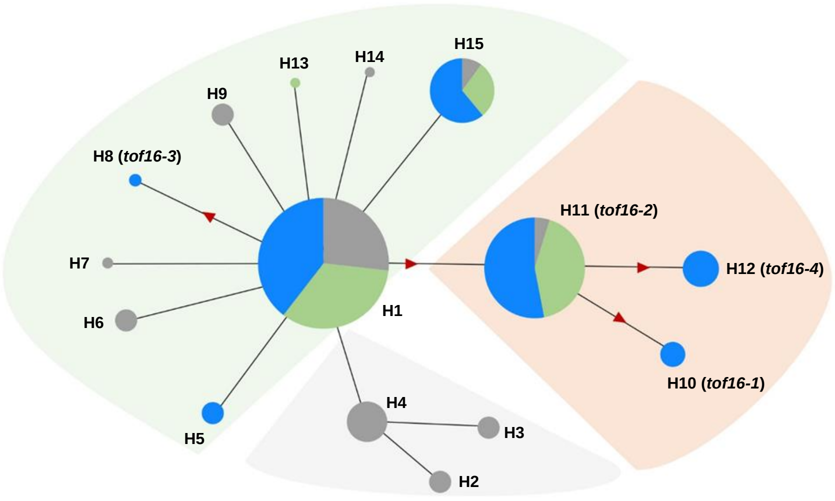

Supplementary Fig. 22 Haplotypes and their origins of *Tof16*.
a Haplotypes of *Tof16*. **b** haplotype origins of *Tof16*. Grey color represented the wild soybeans, green color represented the landraces, blue color represented the improved cultivars. Haplotypes was extracted from the 1624 panel of 256 wild soybeans, 620 landraces and 748 improved cultivars.

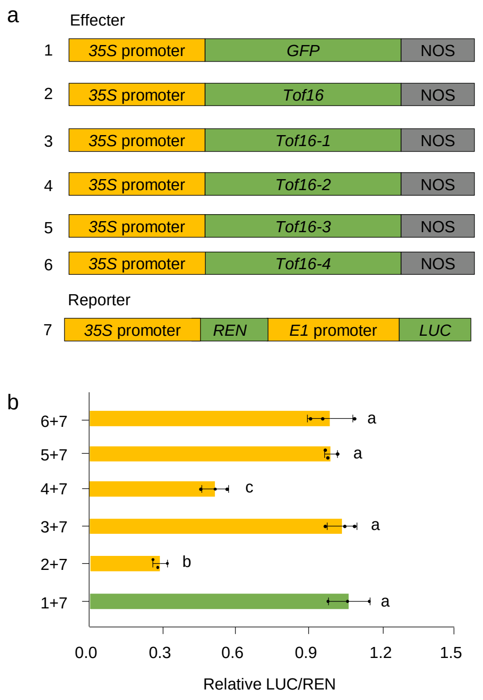

Supplementary Fig. 23 Transient assay of different alleles of *Tof16*.
a Constructs used for the transient transfection assay. **b** Luciferase activity under control of *E1* promoter showing the results from three independent replications. Values are mean ± s.e.m. (n = 3 biologically independent samples), the value of each replication was represented by a dot. The presence of the same lowercase letter above the histogram bars denotes nonsignificant differences across each panel ($P > 0.05$). One-way ANOVA was used to generate the P values. Source data are provided as a Source Data file.

a

Haplotype	SNP Pos.	143	217	272	319	370del	409_410del	518	559	560	588_597del	677	827	838	920	941	1194 TE ins	1282del	1339	1343	1352del	1364del	1441	1675	1796	1843	1847	1864	1976	2134	Number
	J / AA Change	R48T	R73G	P91R	N107H	R124fs	N137fs	Q173R	S187G	S187N	S196fs	P226L	K276M	P280T	R307M	D314G		M428fs	G447S	S448I	S451X	P455fs	P481S	G559C	N599S	A615S	I616K	H622D	L659R	D712N	
**H1**	***J***		G																												42
**H2**	***j-8-2***	C	G					G		A																					3
**H3**	***j-8-1***	C	G					G																							15
**H4**			G	G					G				T			G															1
**H5**			G		C																										11
**H6**	***j-3***		G			-									T																2
**H7**	***j-6***		G				-								T																1
**H8**			G					G						A																	2
**H9**			G																											A	1
**H10**			G					G																							77
**H11**			G						G			T	T			G															1
**H12**			G						G				T			G															5
**H13**			G										T			G							T	T	G						4
**H14**			G										T			G							T		G						6
**H15**			G										T			G							T								3
**H16**			G										T			G															1
**H17**	***j-1***		G												T						-										1
**H18**	***j-10***		G												T							-									2
**H19**			G												T											T					4
**H20**			G												T														G		2
**H21**	***j-11***		G												T																749
**H22**			G												A				A												6
**H23**			G																	T											1
**H24**			G																									G			1
**H25**	***j-9 (e6)***																TE														1
**H26**	***j-2***										-																				5
**H27**	***j-4***																	-													8
**H28**	***J***																														426
**REF.W82**		G	A	C	A	A	AA	A	A	G	TACAAGTAAG	C	A	C	G	A	T	A	G	G	C	C	C	G	A	G	T	C	T	G	

b

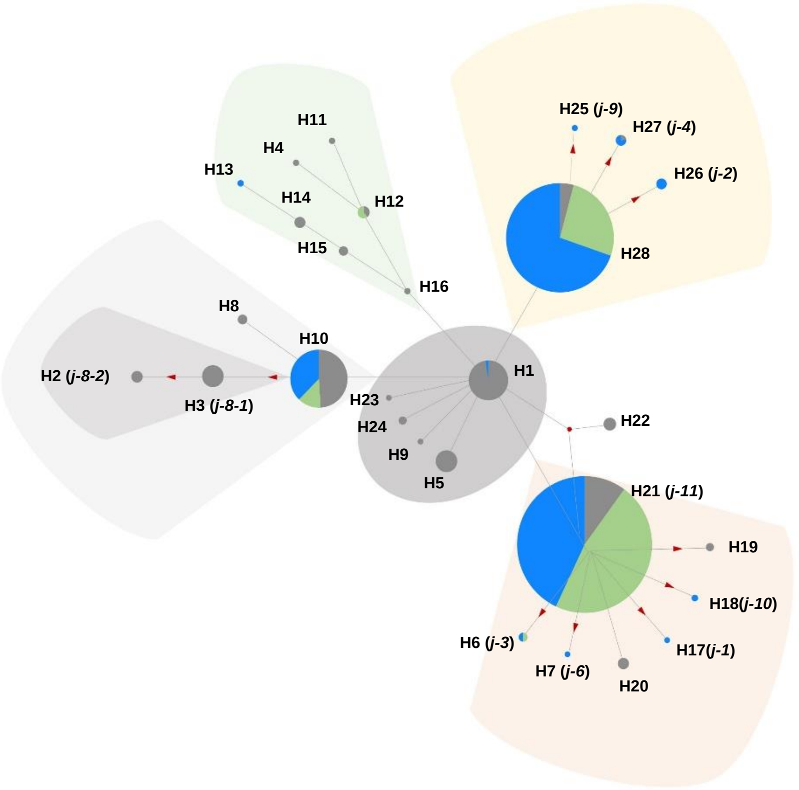

Supplementary Fig. 24 Haplotypes and their origins of *J*.
a Haplotypes of *j*. **b** haplotype origins of Tof11. Grey color represented the wild soybeans, green color represented the landraces, blue color represented the improved cultivars. Haplotypes was extracted from the 1624 panel of 256 wild soybeans, 620 landraces and 748 improved cultivars.

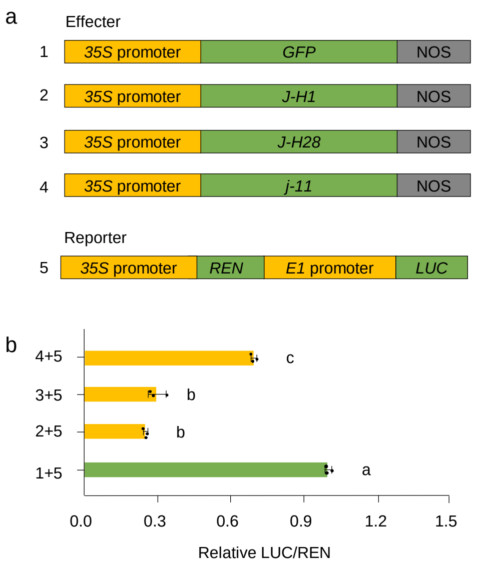

Supplementary Fig. 25 Luciferase activity of J proteins with a nonsynonymous SNP on suppression of the *E1* promoter in *Arabidopsis* transient assays.
a Constructs used for the transient transfection assay. **b** Luciferase activity under control of *E1* promoter showing the results from three independent replications. The value of each replication was represented by a dot. The presence of the same lowercase letter above the histogram bars denotes nonsignificant differences across each panel ($P > 0.05$). One-way ANOVA was used to generate the P values. Source data are provided as a Source Data file.

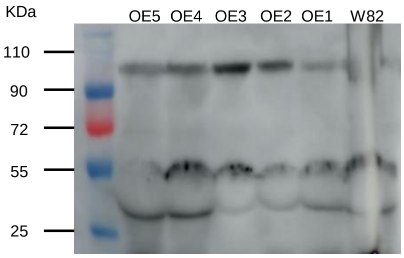

Supplementary Fig. 26 Source data of full gel blot images for supplementary figure 12a.

Supplementary Table 1. Predicted genes in *Tof16* mapping region according to W82 reference genome.

Name	Function
Glyma.16G017200	Inorganic pyrophosphatase
Glyma.16G017300	Serine/threonine-protein kinase 51-related
Glyma.16G017400	MYB-related transcription factor LHY
Glyma.16G017500	Flavanone 3-dioxygenase
Glyma.16G017600	Protein curvature thylakoid 1B
Glyma.16G017700	Transcription factor bHLH57-related
Glyma.16G017800	Cation efflux protein/Zinc transporter
Glyma.16G017900	Late embryogenesis abundant protein
Glyma.16G018000	Pseudo-response regulator 5
Glyma.16G018100	SURFEIT LOCUS PROTEIN 2
Glyma.16G018200	Predicted RNA-binding protein
Glyma.16G018300	Pyruvate dehydrogenase E1 component alpha subunit

Supplementary Table 2. The distribution of the various alleles of the *Tof16* locus in 329 accessions.

Alleles of *Tof16*	Number of accessions	Proportion
Tof16	115	65.00%
tof16-1	9	2.74%
tof16-2	88	26.75%
tof16-3	3	0.91%
tof16-4	15	4.56%

Detailed allelic information of each accessions, please see Supplementary Data 3

Supplementary Table 3. The distribution of the various alleles of the *J* locus in the 329 accessions.

Alleles of *J*	Number of accessions	Proportion
J	151	45.90%
j-1	2	0.61%
j-2	5	1.52%
j-4	4	1.22%
j-6	3	0.91%
j-8-1	2	0.61%
j-8-2	3	0.91%
j-9	1	0.30%
j-10	2	0.61%
j-11	156	47.42%

Detailed allelic information of each accessions, please see Supplementary Data 3

Supplementary Table 4. The frequency of loss-of-function alleles of *tof16* and *j* in 329 low latitude accessions.

Alleles	Numbers of accesions	Total accessions	Propotions
J/TOF16	33	165	20%
j/TOF16	48	165	29%
J/tof16	27	165	16%
j/tof16	57	165	35%
j, tof16, j/tof16	132	165	80%

Detailed allelic information of each accessions, please see Supplementary Data 4

Supplementary Table 5. Primers used in this study.

Primer name	Forward (5'-3')	Reverse (5'-3')	Enzyme	Purpose
E1-qRT	CACTCAAATTAAGCCCTTTCA	TTCATCTCCTCTTCATTTTTGTTG		Primers for qRT-PCR
GmFT2a-qRT	ATCCCGATGCACCTAGCCCA	ACACCAAACGATGAATCCCCA		
GmFT5a-qRT	AGCCCGAACCCTTCAGTAGGGA	GGTGATGACAGTGTCTCTGCCC		
J-qRT	CGTGCCTCATAACCGAAGAT	CTCGTTACATGACATACTCC		
Tof16--qRT	CGTCAGGGAAGATCTGAAGC	ATGCAAAAACCAAAGGTTGC		
Tublin-qRT	TCTTGGACAACGAAGCCATCT	GGTGAGGGACGAAATGATCT		
Tof16-6HA	ATGGACGCATACTCCTCCG	AGTCGAAGTCTCCCCTTCCAA		Primers for gene cloning
Tof16-clone	TAGCAGTTTCTGGTTCTGTTTT	AATTACTTGATGTTCATGGCTC		
E1-promoter	TTGGTTTATGATTCAAGCCGAC	GTTGGAAGAGATGAATAGGGTC		
Tof16-T1	GTCAAGGGAACGATGGACAGA	AAACCCTCTGTCCATCGTTCCCT		Primers for CAS9
Tof6-cas9-	GAGAAAATTGAAGCGAACTGCG	CCCATTGTTGCTAGAACCCC		
EF1b-CHIP	GTTGAAAAGCCAGGGGACA	TCTTACCCCTTGAGCGTGG		Primers for ChIP-PCR
E1-P1	ATTTTGACTGTGGTATGTGACTG	AGAGATAATCGGGAGAAAAACA		
E1-P2	CCCAGTAACGTGGCCAATT	AAATAGTGAAAATCCGGTGACG		
E1-P3				
M1	TTTAGAAGAGAAGAAAGTCCC	GTATGTTGGAATCAGCGTTA	Dra I	Primers for mapping in Chr16
M2	TAGGGTAAGAAAGGGAGAAA	TGACATATGACCTGATAAAAAAT	Dra I	
M3	ATGTTGGTTATGTGGTTTCTTT	CAATCTTATAAAATTGGCTTTTT	Dra I	
M4	GATCAAATATGTTAAGAAATGCG	CAGAACTTCCACCTCATCG	Dpn I	
M5	TAGACAAATTGGTTCCCCTCCT	GAACTGTAGTCACTGATGCA	Bfa I	
M6	AAGCAGCACTCTGAGCACCA	GAAGCAAGTAAAGGGCGGTT	Hae III	
M7	TTCTCATCTCGTTACATTA	ATCCTTCATCAAAACATT	Dra I	

Supplementary Table 6. Resequecing data information of 329 soybean accessions.

	SNP				Indels				SVs			
Group	Total	Intron	Intergenic	Exon	Total	Intron	Intergenic	Exon	DUP	DEL	INS	INV
Total	31,925,948	2,678,267	28,076,106	1,171,575	3,266,897	491,085	2,695,447	80,365	704	7,160	3,781	497
Soja_110	27,640,938	2,484,444	24,078,388	1,078,106	2,655,645	424,107	2,166,040	65,498	431	3,939	2,361	272
Landrace_45	5,990,968	539,120	5,187,563	264,285	405,278	72,750	318,559	13,969	486	2,262	1,091	261
Cultivar_174	10,958,694	891,580	9,660,895	406,219	1,022,217	163,748	830,664	27,805	543	3,666	1,990	310

DUP, Tandem Duplications; DEL, Deletions; INS, Insertions; INV, Inversions.
