# Supplementary material for: Genetic basis and adaptation trajectory of soybean from its temperate origin to tropics
Source: Nat Commun. 2021 Sep 14;12:5445. doi: 10.1038/s41467-021-25800-3 (PMC8440769; doi:10.1038/s41467-021-25800-3)
Supplement: Supplementary file 6 — Description of Additional Supplementary Files [file 41467_2021_25800_MOESM6_ESM.docx]

**Description of Additional Supplementary Files**

File name: Supplementary Data 1
Description: The 329 requenced aceessions.

File name: Supplementary Data 2
Description: Haplotypes of *Tof16* and *J*.

File name: Supplementary Data 3
Description: Haplotypes of *E1*, *Tof16* and *J* in 329 accessions.

File name: Supplementary Data 4
Description: Allellic information of *J* and *Tof16* in low latitude accessions.
