# Supplementary material for: Genetic basis and adaptation trajectory of soybean from its temperate origin to tropics
Source: Nat Commun. 2021 Sep 14;12:5445. doi: 10.1038/s41467-021-25800-3 (PMC8440769; doi:10.1038/s41467-021-25800-3)
Supplement: Supplementary file 7 — Reporting Summary [file 41467_2021_25800_MOESM7_ESM.pdf]

nature portfolio

Corresponding author(s): DBPR and NCOMMS-21-21681A

Last updated by author(s): 25/8/21

Reporting Summary

Nature Portfolio wishes to improve the reproducibility of the work that we publish. This form provides structure for consistency and transparency in reporting. For further information on Nature Portfolio policies, see our Editorial Policies and the Editorial Policy Checklist.

Statistics

For all statistical analyses, confirm that the following items are present in the figure legend, table legend, main text, or Methods section.

n/a	Confirmed	
☐	☒	The exact sample size (*n*) for each experimental group/condition, given as a discrete number and unit of measurement
☐	☒	A statement on whether measurements were taken from distinct samples or whether the same sample was measured repeatedly
☐	☒	The statistical test(s) used AND whether they are one- or two-sided<br>*Only common tests should be described solely by name; describe more complex techniques in the Methods section.*
☐	☒	A description of all covariates tested
☐	☒	A description of any assumptions or corrections, such as tests of normality and adjustment for multiple comparisons
☐	☒	A full description of the statistical parameters including central tendency (e.g. means) or other basic estimates (e.g. regression coefficient) AND variation (e.g. standard deviation) or associated estimates of uncertainty (e.g. confidence intervals)
☐	☒	For null hypothesis testing, the test statistic (e.g. *F*, *t*, *r*) with confidence intervals, effect sizes, degrees of freedom and *P* value noted<br>*Give P values as exact values whenever suitable.*
☐	☒	For Bayesian analysis, information on the choice of priors and Markov chain Monte Carlo settings
☐	☒	For hierarchical and complex designs, identification of the appropriate level for tests and full reporting of outcomes
☒	☐	Estimates of effect sizes (e.g. Cohen's *d*, Pearson's *r*), indicating how they were calculated

Our web collection on statistics for biologists contains articles on many of the points above.

Software and code

Policy information about availability of computer code

Data collection	The sequencing reads were undertaken with Illumina HiSeq X Ten system. No software for data collection was used.
Data analysis	GraphPad Prism v8.0.1, GWAS was performed using the EMMAX;For resequencing data analysis, BWA 0.6.1-r104 was used to align paired-end reads to reference genome Williams 82, SAMtools v0.1.18 (http://picard.sourceforge.net.) were used to sort and filter the mapping results, Genome Analysis Toolkit GATKv3.7-0 to perform whole-genome SNP calling, fillGenotype (http://202.127.18.228/fimg/down.php) was used for imputation for all SNPs; SNP annotation was carried out based on the Williams 82 genome using snpEff v.3.1 software;The remaining SNPs were used to construct a neighbor-joining tree with MEGA v6.06 software and were visualized with the online tool iTOL (https://itol.embl.de). PCA was performed with this SNP set with the smartpca program in the EIGENSOFT v.5.0.1 package.

For manuscripts utilizing custom algorithms or software that are central to the research but not yet described in published literature, software must be made available to editors and reviewers. We strongly encourage code deposition in a community repository (e.g. GitHub). See the Nature Portfolio guidelines for submitting code & software for further information.

Data

Policy information about availability of data

All manuscripts must include a data availability statement. This statement should provide the following information, where applicable:

- Accession codes, unique identifiers, or web links for publicly available datasets
- A description of any restrictions on data availability
- For clinical datasets or third party data, please ensure that the statement adheres to our policy

The sequencing data (329 accessions) used in this study have been deposited into the NCBI database under accession number PRJNA728982 (https://

www.ncbi.nlm.nih.gov/bioproject/PRJNA728982/). The previously reported sequence data were deposited into the NCBI database under accession number PRJNA394629 and the GSA database in BIG Data Center under accession number PRJCA000205 and PRJCA001691.

Field-specific reporting

Please select the one below that is the best fit for your research. If you are not sure, read the appropriate sections before making your selection.

☒ Life sciences ☐ Behavioural & social sciences ☐ Ecological, evolutionary & environmental sciences

For a reference copy of the document with all sections, see nature.com/documents/nr-reporting-summary-flat.pdf

Life sciences study design

All studies must disclose on these points even when the disclosure is negative.

Sample size	The sample size was determined according to the reports in the related research subjects. For phenotype evaluation, at least ten individual plants were analyzed per accession. No statistical methods were used to predetermine sample sizes.
Data exclusions	No data were excluded from our analyses.
Replication	Association analysis was repeated at least twice. Expression and phenotype were repeated three times. This information is shown in figure legends.
Randomization	The samples in all the expression and phenotyping investigation were randomly sampled.
Blinding	For molecular biology experiments, bias could not be introduced since samples were treated identically and collected randomly. Blind was not possible as the author who performed the experiment also analyzed the data. Investigation of agronomic traits were performed without prior knowledge of the result, blind was not applied.

Reporting for specific materials, systems and methods

We require information from authors about some types of materials, experimental systems and methods used in many studies. Here, indicate whether each material, system or method listed is relevant to your study. If you are not sure if a list item applies to your research, read the appropriate section before selecting a response.

Materials & experimental systems

n/a	Involved in the study	
☐	☒	Antibodies
☒	☐	Eukaryotic cell lines
☒	☐	Palaeontology and archaeology
☒	☐	Animals and other organisms
☒	☐	Human research participants
☒	☐	Clinical data
☒	☐	Dual use research of concern

Methods

n/a	Involved in the study	
☒	☐	ChIP-seq
☒	☐	Flow cytometry
☒	☐	MRI-based neuroimaging

Antibodies

Antibodies used	The anti-HA antibody (Abcam, ab18181, 1:5000 dilution for Immunoblot)
Validation	The anti-HA antibody (ab18181) was already commercialized and has been validated to work in soybean (Lu et al., Nat. Genet. 2020, 52, 428-436. https://doi.org/10.1038/s41588-020-0604-7)
